# Crystal Structures of the Global Regulator DasR from *Streptomyces coelicolor*: Implications for the Allosteric Regulation of GntR/HutC Repressors

**DOI:** 10.1371/journal.pone.0157691

**Published:** 2016-06-23

**Authors:** Simon B. Fillenberg, Mario D. Friess, Samuel Körner, Rainer A. Böckmann, Yves A. Muller

**Affiliations:** 1 Division of Biotechnology, Department of Biology, Friedrich-Alexander University Erlangen-Nuremberg, Henkestr. 91, D-91052 Erlangen, Germany; 2 Computational Biology Group, Department of Biology, Friedrich-Alexander University Erlangen-Nuremberg, Staudtstr. 5, D-91058 Erlangen, Germany; University of Washington, UNITED STATES

## Abstract

Small molecule effectors regulate gene transcription in bacteria by altering the DNA-binding affinities of specific repressor proteins. Although the GntR proteins represent a large family of bacterial repressors, only little is known about the allosteric mechanism that enables their function. DasR from *Streptomyces coelicolor* belongs to the GntR/HutC subfamily and specifically recognises operators termed DasR-responsive elements (*dre*-sites). Its DNA-binding properties are modulated by phosphorylated sugars. Here, we present several crystal structures of DasR, namely of dimeric full-length DasR in the absence of any effector and of only the effector-binding domain (EBD) of DasR without effector or in complex with glucosamine-6-phosphate (GlcN-6-P) and *N*-acetylglucosamine-6-phosphate (GlcNAc-6-P). Together with molecular dynamics (MD) simulations and a comparison with other GntR/HutC family members these data allowed for a structural characterisation of the different functional states of DasR. Allostery in DasR and possibly in many other GntR/HutC family members is best described by a conformational selection model. In ligand-free DasR, an increased flexibility in the EBDs enables the attached DNA-binding domains (DBD) to sample a variety of different orientations and among these also a DNA-binding competent conformation. Effector binding to the EBDs of DasR significantly reorganises the atomic structure of the latter. However, rather than locking the orientation of the DBDs, the effector-induced formation of β-strand β* in the DBD-EBD-linker segment merely appears to take the DBDs ‘on a shorter leash’ thereby impeding the ‘downwards’ positioning of the DBDs that is necessary for a concerted binding of two DBDs of DasR to operator DNA.

## Introduction

Bacteria are highly versatile microorganisms that are able to swiftly adapt their metabolism to environmental changes [1]. Mechanisms that rapidly alter bacterial gene transcription rates and thereby the composition of the bacterial proteome are of central importance for this adaptation process [2]. In prokaryotes, various transcription regulator proteins exist that bind to specific operator DNA sequences and function as one-component signal transduction elements. Their ability to either facilitate or abrogate gene transcription is directly modulated *via* the interaction with low molecular weight effector molecules [2, 3].

The GntR protein family constitutes one of the largest families of prokaryotic transcription regulators (Pfam family: PF00392) [4–6]. Members of this family share a common N-terminal winged-helix-turn-helix (wHTH) DNA-binding domain (DBD), but display different C-terminal effector-binding and oligomerisation domains (EBD). The diversity of the latter domain justifies a further division of the GntR family, with GntR/FadR, -/HutC, -/MocR and -/YtrA representing the most important subfamilies [6, 7]. Recent crystal structures of operator-derived DNA segments in complex with either full-length GntR proteins (i.e. FadR from the GntR/FadR and NagR from the GntR/HutC subfamily) or with isolated DBDs (AraR from the GntR/AraR subfamily) revealed a shared DNA-binding mode among the different GntR subfamilies and highlighted common features in sequence-specific DNA recognition [6, 8–11]. This binding mode is characterised by a juxtaposed binding of two DBDs to an about 15 base-pair-long contiguous major groove segment and clearly differs from that observed in non-GntR-type wHTH-domain-containing bacterial repressors such as, for example, the MarR family [12]. However, in contrast to the DBDs, the EBDs of the GntR family members display highly diverse folds, and, as a consequence, effector recognition differs considerably between the GntR subfamilies [6].

GntR/HutC is, next to GntR/FadR, the second most populated GntR subfamily and comprises about 30% of all GntR transcription factors [6]. The subfamily is named after the histidine utilisation operon regulator HutC from *Pseudomonas putida* [13]. Structural studies corroborated a prior prediction, namely that the EBD fold in these transcription regulators resembles that of monomeric chorismate lyase UbiC from *Escherichia coli* [14–16]. Moreover, these studies showed that the UbiC transcription regulator-associated (UTRA) domain also serves as a dimerisation domain, and that the dimerisation mode is strictly conserved throughout the GntR/HutC subfamily [14–16]. Although numerous crystal structures of individual EBDs of GntR/HutC family members are available from the protein data bank, only a few of these describe full-length repressors comprising both the DNA- and effector-binding domain [17]. Besides full-length PhnF from *Mycobacterium smegmatis* [18] and NagR (previously known as YvoA) from *Bacillus subtilis* [11, 16], full-length crystal structures are available for YydK from *B*. *subtilis* (PDB-ID 3BWG; UniProtKB Q45591) and for an unnamed regulator from *Streptomyces avermitilis* (PDB-ID 3EET; UniProtKB Q82IF8) [17, 19].

Only in the case of the GntR/HutC family members PhnF and NagR it was so far possible to relate observations from multiple crystal structures to different functional states [11, 16, 18]. The crystal structure of full-length NagR from *B*. *subtilis* with sulphate molecules bound in the effector-binding site was determined first followed by sulphate-bound PhnF from *M*. *smegmatis* and by crystal structures of NagR bound to an idealised operator DNA sequence as well as of NagR in complex with the putative effector molecules glucosamine-6-phosphate (GlcN-6-P) and *N*-acetylglucosamine-6-phosphate (GlcNAc-6-P) [11, 16, 18]. The structures are consistent with a model wherein ligand binding to PhnF and NagR repositions two particular α-helices in the EBDs and at the same time stabilises segments that appear to display high flexibility in the absence of ligands [11, 15, 18]. A comparison between the DNA-bound and the sugar-bound structures of NagR suggested that a so-called ‘jumping jack’-like movement of the DBDs characterises the allosteric mechanism that modulates the DNA-binding affinity in NagR [11, 16]. It remains, however, unclear whether the extraordinary large displacement of the DBDs by 70 Å also occurs in other GntR/HutC family members, and to what extent this constitutes a hallmark of the allosteric regulation of GntR/HutC repressors.

DasR from the Gram-positive soil-dwelling bacterium *Streptomyces coelicolor* is one of the functionally best characterised members of the GntR/HutC family. The DNA-binding sites for DasR are *cis*-regulatory elements with a length of 16 base pairs and are termed DasR-responsive elements (*dre*-sites) [20]. Gene repression by DasR is abrogated by several effectors such as, for example, GlcN-6-P [21, 22]. In addition to the regulation of the *N*-acetylglucosamine-related catabolic genes, DasR also participates in global processes like cell development and secondary metabolite biosynthesis [21, 23, 24]. Due to the presence of an extraordinary large number of putative *dre*-sites in *S*. *coelicolor*, DasR was named a global regulator [23]. The fact that DasR also regulates genes involved in the synthesis of antibiotic compounds renders DasR of biotechnological interest [23–25].

Here, we present crystal structures of DasR, namely of ligand-free full-length DasR and of only the EBDs in ligand-free form as well as in complex with GlcN-6-P and GlcNAc-6-P. Together with molecular dynamics (MD) simulations we provide a wealth of structural data that suggest that allostery in DasR and possibly in all GntR/HutC family members is best described by a conformational selection model rather than by a two-state allosteric model.

## Results

### Effector binding to DasR engenders distinct structural rearrangements in the effector-binding domain

**Fig 1:**
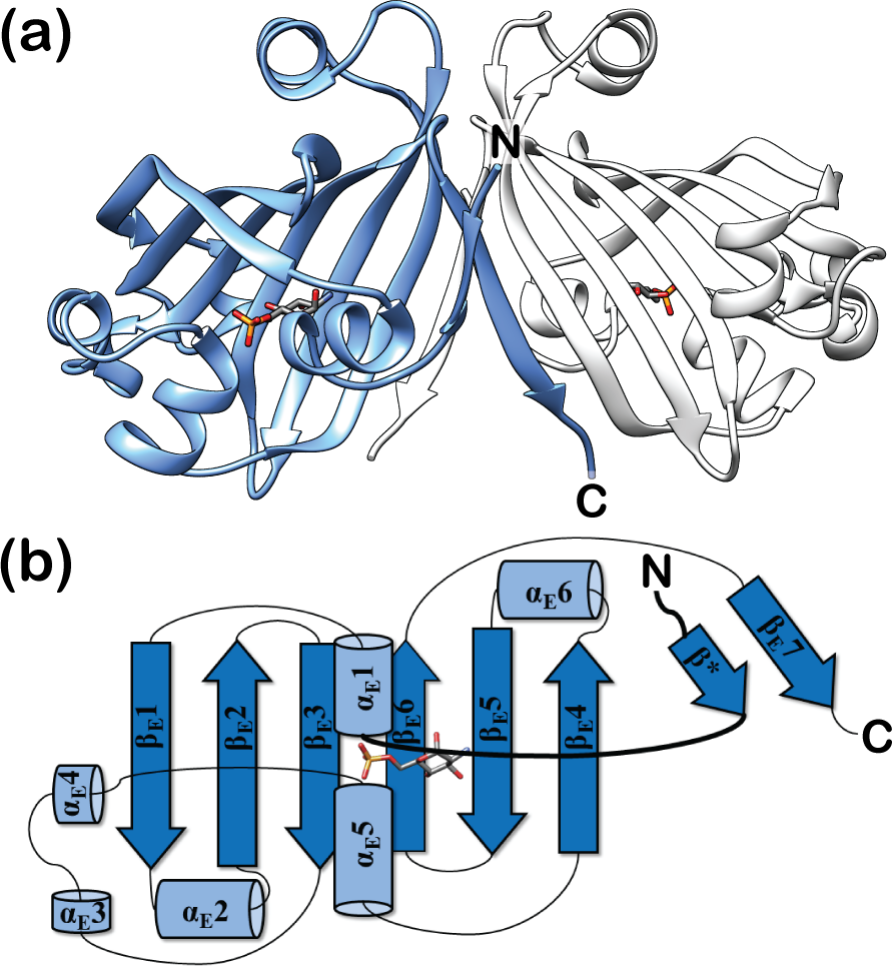
Structure of DasR-EBD in complex with GlcN-6-P. (a) Crystal structure of dimeric DasR-EBD bound to GlcN-6-P in a cartoon representation. The monomers are coloured in blue and light grey, while GlcN-6-P is shown as a stick model. (b) Topology plot of monomeric DasR-EBD in complex with GlcN-6-P. Secondary structure elements are displayed as blue cylinders (α-helices) and arrows (β-strands). The linker segment between the DBD and EBD is highlighted in bold. Due to the highly similar overall conformation, the GlcNAc-6-P-bound DasR-EBD structure is not shown.

The structures of DasR-EBD in complex with GlcN-6-P and GlcNAc-6-P were solved by X-ray crystallography at 1.65 Å and 1.85 Å resolution, respectively, in order to characterise the effector binding of DasR (Table 1). Both structures contain a homodimer in the asymmetric unit, and each effector-binding site is occupied by a phosphorylated sugar molecule (Fig 1).

**Table 1:** Data collection and refinement statistics.

Dataset	DasR-EBD	DasR-EBD	DasR-EBD	DasR
	+ GlcN-6-P	+ GlcNAc-6-P	(ligand-free)	(ligand-free)
PDB-ID	4ZSI	4ZSK	4ZSB	4ZS8
**Data collection** ^a^				
Beamline	BESSY, BL 14.1	BESSY, BL 14.1	BESSY, BL 14.1	BESSY, BL 14.1
Wavelength (Å)	0.91841	0.91841	0.91841	0.91841
Resolution (Å)	35.00–1.65	35.00–1.85	50.00–2.00	50.00–2.60
	(1.71–1.65)	(1.92–1.85)	(2.08–2.00)	(2.69–2.60)
Space group	P3_2_21	P3_2_21	P3_1_21	P4_2_2_1_2
Cell parameters (Å/°)	54.3 / 54.3 / 220.9	54.2 / 54.2 / 222.5	56.6 / 56.6 / 109.0	96.0 / 96.0 / 118.9
	90 / 90 / 120	90 / 90 / 120	90 / 90 / 120	90 / 90 / 90
Total reflections	262387 (25772)	261688 (25611)	56273 (3882)	141168 (13977)
Unique reflections	46439 (4535)	33544 (3282)	14018 (1294)	17696 (1724)
Redundancy	5.7 (5.7)	7.8 (7.8)	4.0 (3.0)	8.0 (8.1)
Completeness (%)	99.8 (99.7)	99.9 (99.5)	98.9 (94.2)	99.9 (99.7)
*I/σ(I)*	21.0 (2.1)	11.7 (1.8)	17.5 (1.4)	21.3 (2.2)
Wilson *B*-value (Å^2^)	23.4	25.6	36.9	55.8
*R*_merge_^b^ (%)	4.6 (81.2)	11.3 (112.2)	5.4 (80.6)	9.2 (103.0)
*R*_meas_^c^ (%)	5.1	12.1	6.3	9.8
CC_1/2_^d^ (%)	99.9 (65.7)	99.7 (68.5)	99.8 (61.2)	99.9 (73.3)
**Structure refinement**				
Resolution (Å)	27.13–1.65	32.30–1.85	44.73–2.00	44.52–2.60
*R*_work_/*R*_free_ (%)^e^	18.97 / 23.19	19.16 / 24.82	21.22 / 25.62	20.10 / 28.50
No. of non-hydrogen atoms	2930	2943	1361	3697
No. of protein residues	330	331	162	461
No. of solvent molecules	279	253	75	32
Additional molecules	2 x GlcN-6-P	2 x GlcNAc-6-P		5 x ethylene glycol
	2 x ethylene glycol	4 x ethylene glycol		
Average *B*-factors (Å^2^)	27.5 (all atoms)	30.2 (all atoms)	42.3 (all atoms)	60.4 (all atoms)
	26.7 (protein)	29.6 (protein)	42.3 (protein)	60.5 (protein)
	35.5 (water)	37.4 (water)	41.0 (water)	51.5 (water)
	26.0 (ligands)	30.2 (ligands)		64.9 (ligands)
Ramachandran favoured (%)	98.2	98.5	98.0	96.1
Ramachandran outliers (%)	0.0	0.0	0.0	1.5
R.m.s. deviations				
Bond lengths (Å)	0.007	0.007	0.009	0.008
Bond angles (°)	1.096	1.104	1.101	1.217

^a^ Values for the highest resolution shell are listed in parentheses.

^b^ *R*_merge_ = Σ|*I*—<*I*>|/Σ*I*, where *I* is the integrated intensity of a given reflection.

^c^ *R*_meas_ is the multiplicity weighted merging *R*-factor.

^d^ Correlation coefficient between two random halves of the dataset as described by Karplus and Diederichs[36], and calculated using XDS [35].

^e^ *R*_work_ = Σ||*F*_obs_|—|*F*_calc_||/Σ|*F*_obs_|. *R*_free_ was calculated using 5–10% of data excluded from refinement.

**Fig 2:**
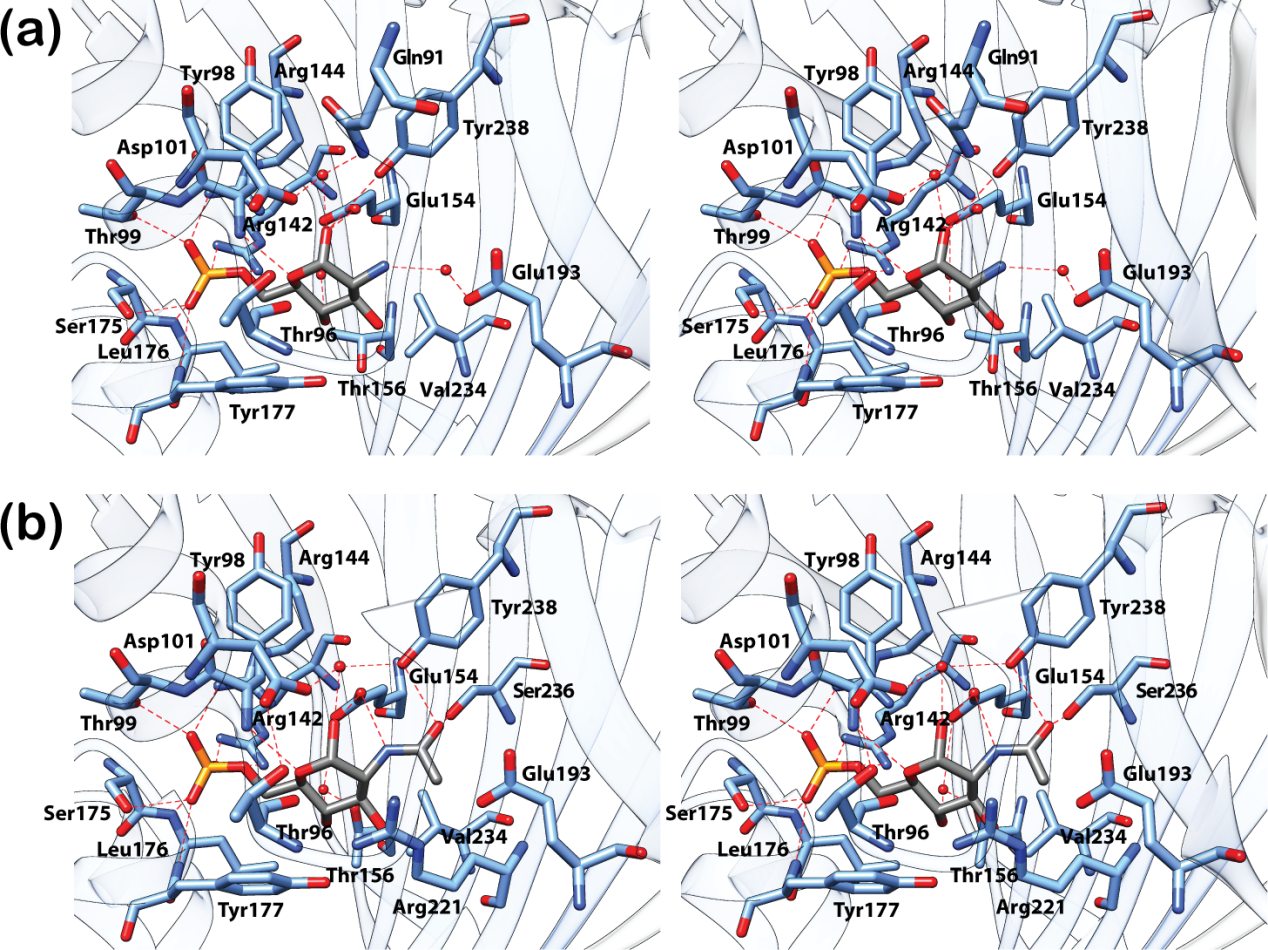
Closeup view of the effector-binding site of DasR-EBD. The stereo views show the interaction of DasR-EBD with the α-anomeric configuration of (a) GlcN-6-P and (b) GlcNAc-6-P. GlcN-6-P, GlcNAc-6-P and the interacting protein residues are presented as stick models and water molecules are depicted as red spheres. In the sugar molecules, the phosphor, oxygen, nitrogen and carbon atoms are coloured in yellow, red, dark blue and grey, respectively.

As previously observed for NagR, the phosphate moieties of GlcN-6-P and GlcNAc-6-P are ‘sandwiched’ between the N-termini of helices α_E_1 (Thr99) and α_E_5 (Ser175, Leu176, Tyr177) in the effector-bound DasR-EBD structures and are in addition coordinated by two arginines (Arg142 and Arg144) from β-strand β_E_2 (Fig 2). The sugar ring in each effector is recognised *via* direct and water-mediated hydrogen bonds involving residues Tyr98, Glu154, Glu193 and Tyr238 as well as *via* interactions with hydrophobic segments of Ser97, Arg221, Glu232 and Val234. A stacking of the sugar ring against Tyr177 is reminiscent of the interaction of sugars with aromatic side chains as for example observed in sugar transporters such as the LamB [26]. Furthermore, in the complexes of DasR-EBD with GlcN-6-P and GlcNAc-6-P, an additional β-strand termed β* is formed in the linker segment that connects the DBD to the EBD (Fig 1). This β-strand is also observed in effector-bound NagR, but is absent in DNA-bound NagR [11]. Interestingly and different from NagR, one protein chain from each DasR-EBD dimer binds the phosphorylated ligand in an α-anomeric configuration, whereas the second chain preferentially binds the corresponding β-anomer (S1 and S2 Figs). Although differences in the hydrogen-bonding network surrounding carbon C1 exist between the two anomers, these do not lead to differences in the overall conformation of the EBDs since the average pairwise r.m.s. deviation of the Cα-atoms of all four monomers in the two effector-bound complexes is as low as 0.61 Å.

**Fig 3:**
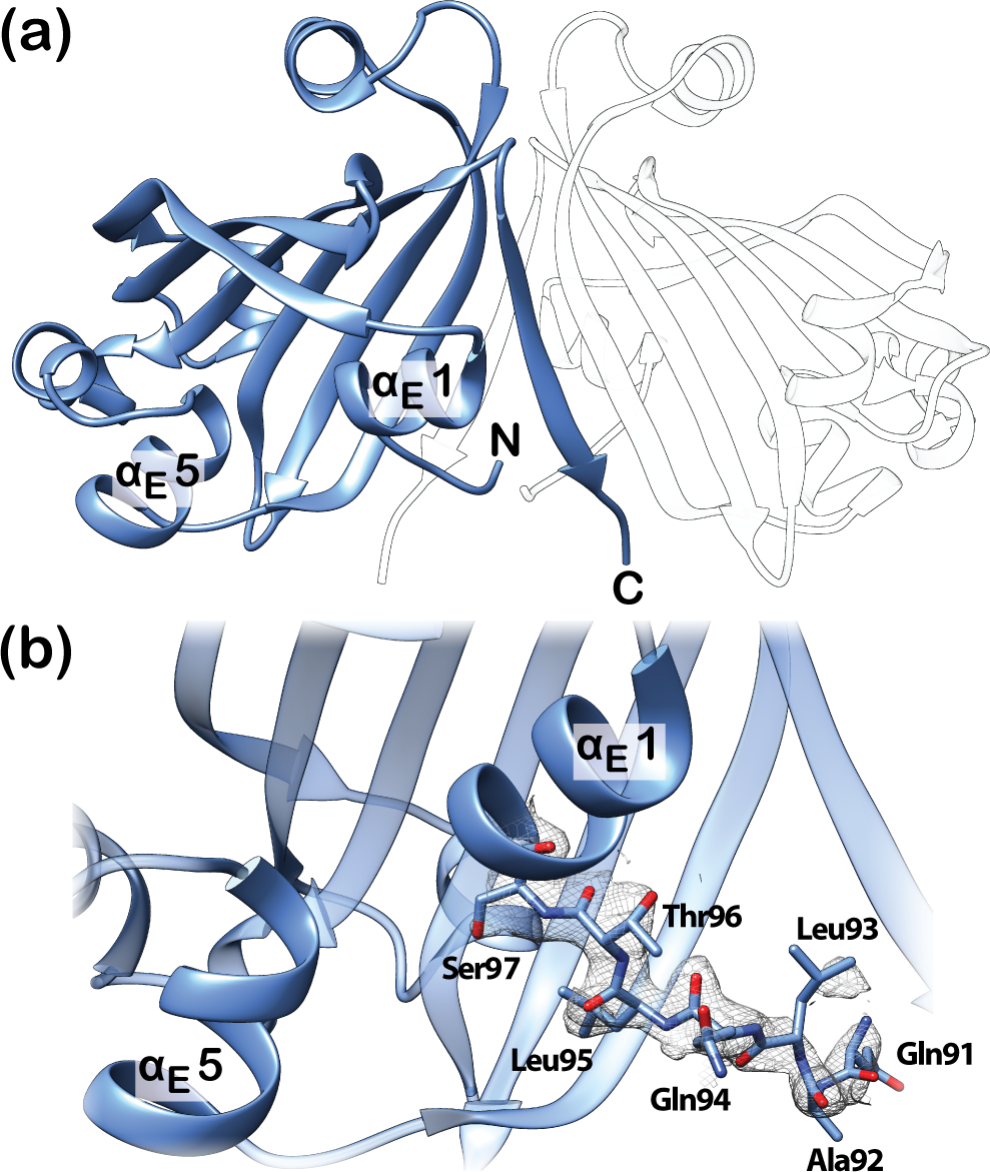
Structure of ligand-free DasR-EBD. (a) Crystal structure of a ligand-free DasR-EBD dimer. Only one monomer is contained in the asymmetric unit (shown in blue) and a second, symmetry-related monomer is shown in transparent white. (b) Closeup view of the effector-binding site and the N-terminal interdomain linker in ligand-free DasR-EBD. Residues 91–97 of the linker segment are shown as stick model with the corresponding *2F*_*o*_*-F*_*c*_ map (grey mesh) contoured at 1 σ to highlight the well-defined conformation of the main chain of this segment.

**Fig 4:**
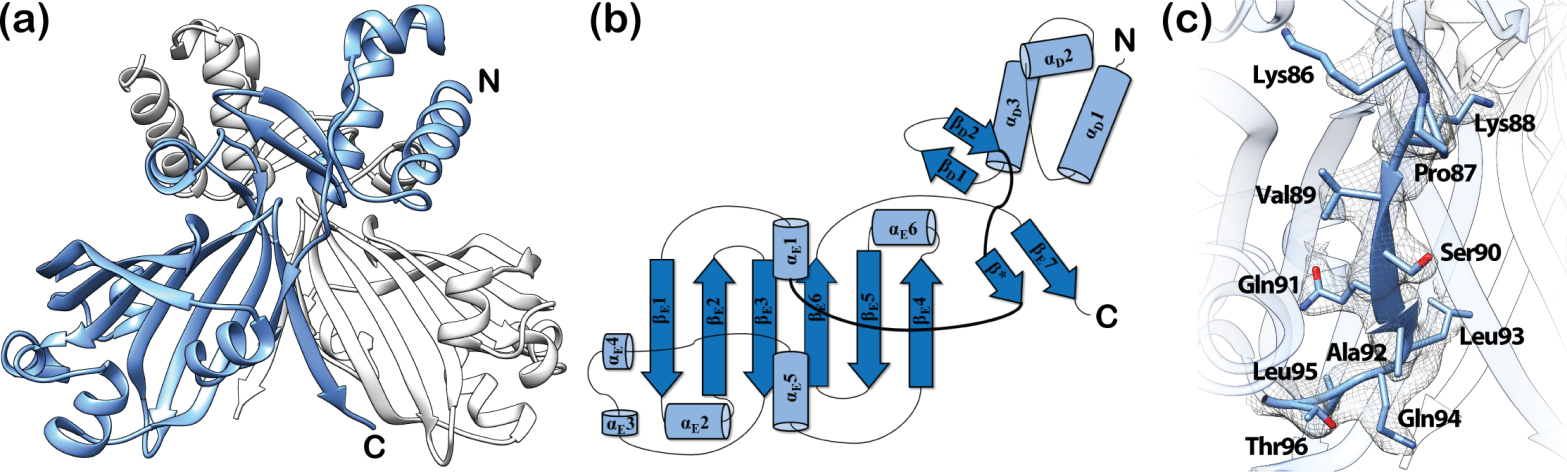
Structure and topology plot of ligand-free full-length DasR. (a) DasR dimer without DNA or a potential effector molecule with the monomers coloured in blue and light grey. (b) Topology plot of full-length DasR in the ligand-free state. Secondary structure elements are displayed as blue cylinders (α-helices) and arrows (β-strands). The linker segment between the DBD and EBD is highlighted in bold. (c) Closeup view of the linker segment between the DBD and EBD in ligand-free DasR. Residues 86–96 of the linker segment are shown as stick model with the corresponding *2F*_*o*_*-F*_*c*_ electron density (grey mesh) contoured at 1 σ to emphasize the well-ordered secondary structure in this region.

In addition to the above-mentioned complexes, the crystal structures of ligand-free DasR-EBD (Fig 3A) and ligand-free full-length DasR (Fig 4A) could also be determined at resolutions of 2.0 Å and 2.6 Å, respectively (Table 1). While full-length DasR encompasses a homodimer in the asymmetric unit, DasR-EBD crystallised with only one monomer in the asymmetric unit and forms a homodimer with a crystallographically related neighbouring monomer. In ligand-free DasR-EBD, the cleft between helices α_E_1 and α_E_5 is devoid of any bound molecule, and, in contrast to effector-bound DasR-EBD, the N-terminal β-strand β* is not formed (Fig 3A). Instead of β*, the corresponding segment forms a loop that is directed towards the C-terminal end of the EBD (Fig 3B). The conformation of this segment resembles that observed in full-length NagR in complex with DNA [11].

In contrast, β-strand β* is present in ligand-free full-length DasR and as a consequence, this ligand-free structure displays an ‘upwards’-directed positioning of the DBDs (Fig 4). A similar ‘upwards’ orientation of the DBDs was so far only observed for effector-bound NagR, and it was inferred that this conformation represents the non-DNA-binding induced conformation of NagR [11]. However, in the case of ligand-free full-length DasR the DBDs are ‘upwards’-directed despite the fact that the effector-binding pocket is empty.

**Fig 5:**
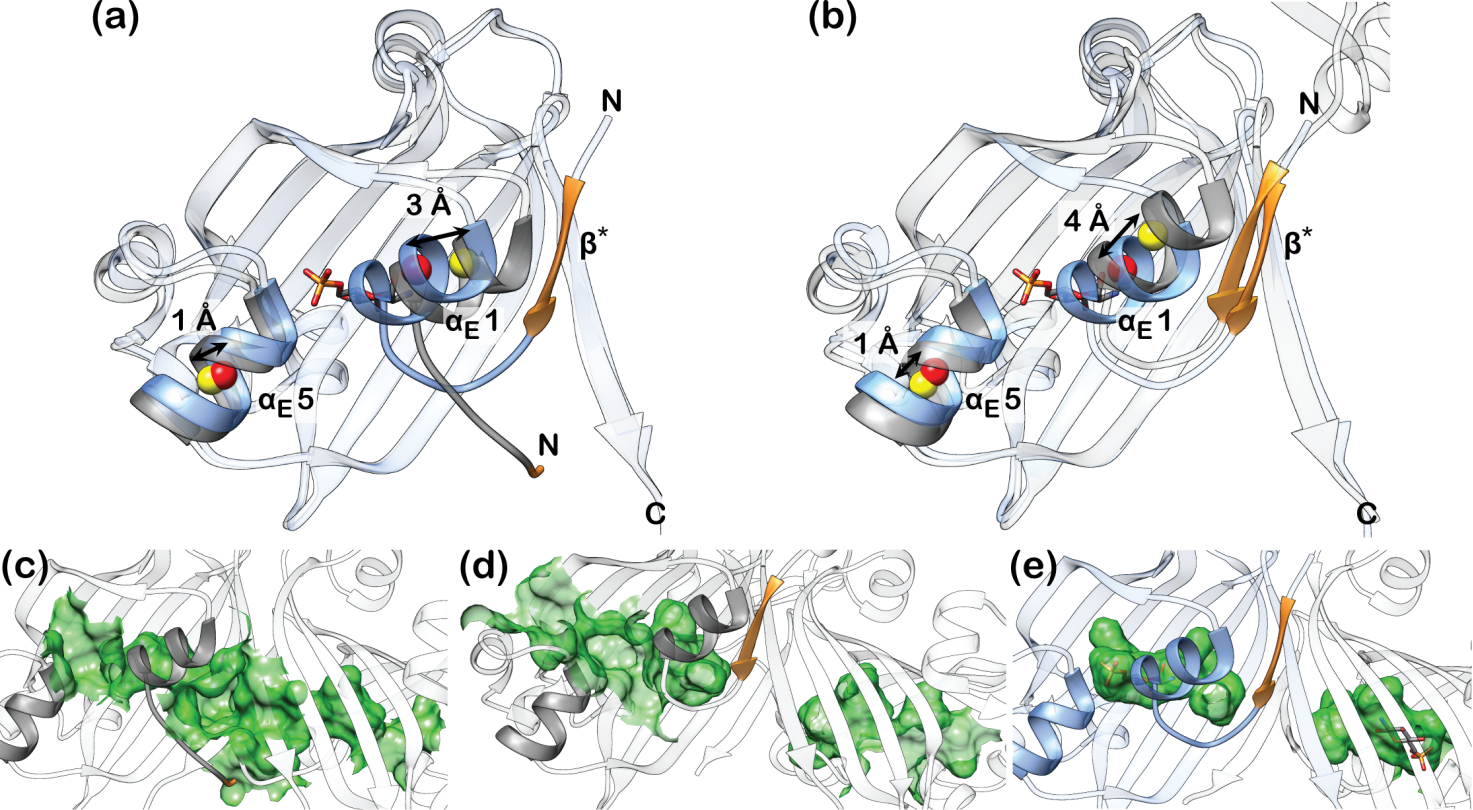
Structural rearrangements in DasR-EBD upon effector binding. Superposition of DasR-EBD in complex with α-anomeric GlcN-6-P and the ligand-free structures of (a) DasR-EBD and (b) full-length DasR. For clarity, only one monomer of the biologically active dimer is displayed. The GlcN-6-P-bound state is shown in blue and the ligand-free state in grey. The regions comprising β-strand β* or the corresponding loop-forming residues are shown in orange. The centres of mass for helices α_E_1 and α_E_5 were calculated with CHIMERA using identical residues in each helix. They are marked as coloured spheres, namely in yellow for the effector-free and in red for the effector-bound structures. The EBD sections of the depicted monomers show pairwise r.m.s. deviations of the Cα-atoms of (a) 1.35 Å and (b) 1.48 Å. (c-e) Comparison of the different sizes of the effector-binding pockets in (c) ligand-free DasR-EBD, (d) ligand-free DasR and (e) DasR-EBD in complex with GlcN-6-P. The mapped binding pocket surfaces are depicted in green.

A structural superposition of all these different DasR structures reveals a characteristic displacement of helices α_E_1 and α_E_5 upon binding of the phosphorylated sugars (Fig 5A and 5B). Helix α_E_5 moves by about 1 Å, while helix α_E_1, which is attached to the linker segment that separates the DBD and EBD, shifts by a distance of about 3–4 Å. Effector binding to DasR reduces the spacing between both helices by 4–5 Å in total. The effector-binding pocket extends across both monomers in ligand-free DasR-EBD and encompasses a total solvent-accessible volume of about 440 Å^3^ (220 Å^3^ per monomer) (Fig 5C; S1 Table). This contiguous pocket becomes segregated into two individual pockets once β-strand β* is formed in each chain in ligand-free dimeric DasR, however, without reducing the size of the individual pockets (about 260 Å^3^ per monomer) (Fig 5D; S1 Table). Binding of the small molecule effectors and the concomitant rapprochement of helices α_E_1 and α_E_5 considerably reduces the volume that is available for GlcN-6-P- and GlcNAc-6-P-binding to a mere 90 Å^3^ per pocket (Fig 5E; S1 Table). Whereas in the ligand-free structures two surface-located openings provide access to the binding pocket in each monomer (Fig 5C and 5D; S1 Table), the binding pockets are completely shielded from the outside in the effector-bound structures (Fig 5E). Altogether, the crystal structures show that clear differences can be identified between the effector-bound and effector-free DasR.

### Ligand-free full-length DasR reveals ‘yet another’ positioning of the DBDs with respect to the EBD dimer core

**Fig 6:**
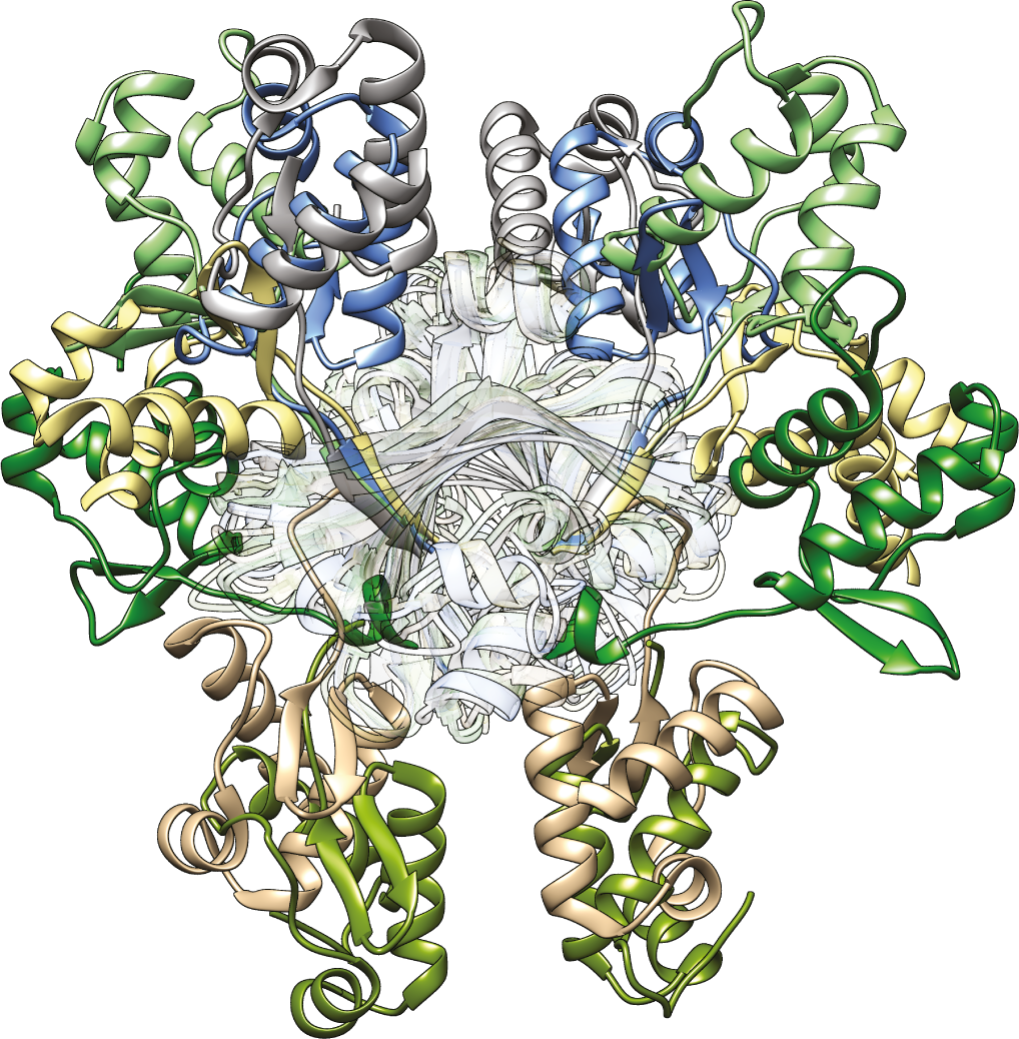
High diversity in the positioning of the DBDs observed among the GntR/HutC transcription factors. Superposition of the crystal structures of ligand-free SauR from *S*. *avermitilis* (DBD in grey, PDB-ID 3EET), ligand-free DasR from *S*. *coelicolor* (blue, PDB-ID 4ZS8), GlcNAc-6-P-bound NagR from *B*. *subtilis* (light green, PDB-ID 4U0W), ligand-free PhnF from *M*. *smegmatis* (light yellow, PDB-ID 3F8M), sulphate-bound NagR (dark green, PDB-ID 2WV0), ligand-free YydK from *B*. *subtilis* (light brown, PDB-ID 3BWG) and NagR in complex with palindromic dsDNA (olive, PDB-ID 4WWC). The prior listing correlates with the clockwise display of the DBDs of the various proteins starting with the DBD of SauR (in grey) at the top of the figure. The superimposed EBDs of the dimeric repressors are located in the centre and rendered transparent. The superimposed EBDs show the following r.m.s. deviations between Cα-positions when compared to the EBD (residues 87–252, chain A) of ligand-free DasR: 1.76 Å (ligand-free SauR), 2.08 Å (GlcNAc-6-P-bound NagR), 1.47 Å (ligand-free PhnF), 2.13 Å (sulphate-bound NagR), 1.94 Å (ligand-free YydK) and 1.65 Å (DNA-bound NagR).

**Fig 7:**
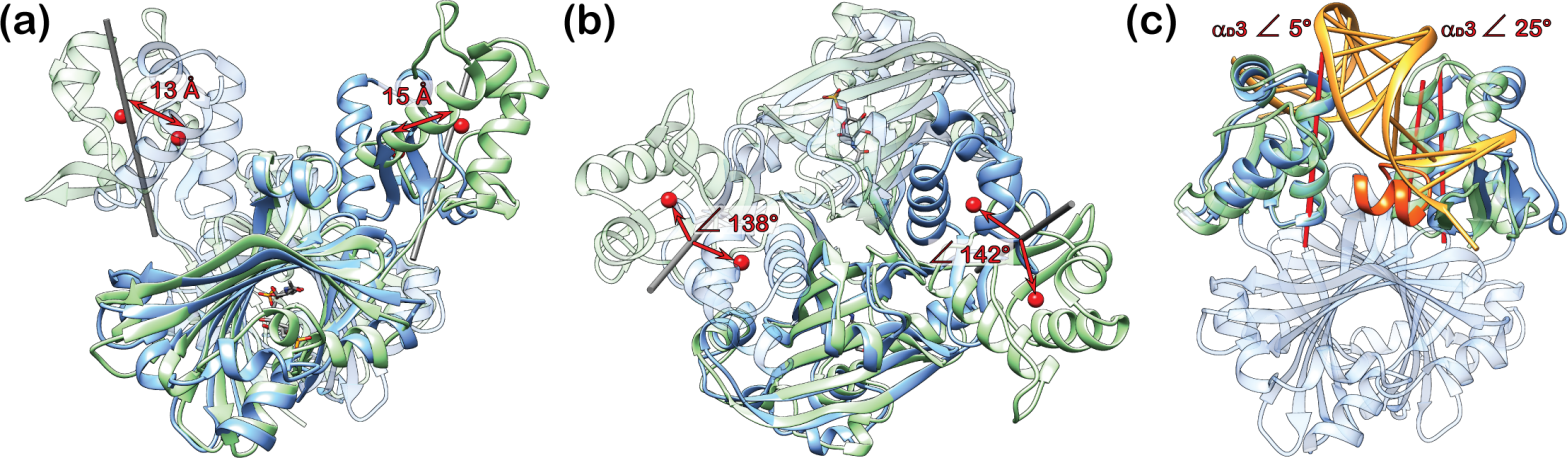
A comparison of ligand-free DasR with the GlcNAc-6-P- and DNA-bound structures of NagR. (a) and (b) Superposition of dimers of ligand-free DasR (blue) and GlcNAc-6-P-bound NagR (green) displayed in a side view (a) and a top view (b). The centres of mass of the DBDs in each dimer were calculated with CHIMERA and are presented as red spheres. The DBDs of DasR and NagR can be oriented identically after applying a rotation of about 140° to either one of the DBDs around the axes indicated in grey (grey rods) and as calculated with program CHIMERA (138°, left DBDs and 142°, right DBDS). (c) Superposition of a ligand-free DasR dimer (blue) with the crystal structure of NagR-DBDs (green) in complex with DNA (orange). Helix α_E_6 of chain A of DasR is coloured in orange red. The helix axes (red rods) of the DNA-recognition helices α_D_3 were calculated with CHIMERA.

The crystal structures of full-length GntR/HutC members that are available from the protein data bank display an unexpected diversity in the orientation of the DBDs with respect to the EBDs (Fig 6). This holds also true for crystal structures from identical members. Thus, a comparison of the DNA- and effector-bound full-length structures of NagR reveals that the DBDs are displaced by about 70 Å between these structures (Fig 6) [11]. At a first glance, the overall positioning of the DBDs in ligand-free full-length DasR resembles that observed in NagR in complex with potential effector molecules. However, also in this case, the centres of mass of the DBDs are shifted by about 13 to 15 Å between both structures (Fig 7A). In addition, each DasR-DBD would have to be rotated by approximately 140° in order to mirror the orientation seen in GlcNAc-6-P-bound NagR (Fig 7B).

Interestingly, the overall pairwise orientation of the DBDs in ligand-free full-length DasR resembles that previously observed in the crystal structure of isolated NagR-DBDs in complex with palindromic dsDNA (Fig 7C) [11]. More specifically, when aligning the DasR-DBD of one monomer with one DNA-bound NagR-DBD, then the DasR-DBD of the second monomer is only slightly tilted with regard to the corresponding second NagR-DBD, which is emphasised by a deviation angle of 25° between the DNA-recognition helices α_D_3 in the two respective DBDs. In theory it seems therefore possible that, upon small conformational adjustments, DasR can possibly bind DNA with ‘upwards’-oriented DBDs, whereas, by contrast, the crystal structure of full-length NagR in complex with DNA clearly showed that in NagR a ‘downwards’ orientation is required for DNA binding [11]. In order to investigate whether or not DasR is able to bind DNA with ‘upwards’-oriented DBDs we performed a number of superposition and molecular dynamics calculations.

### The ‘upwards’ positioning of the DBDs does not allow for a concerted binding of DasR to operator DNA sequences

Initial hints that ligand-free DasR is not able to bind DNA with ‘upwards’-oriented DBDs can be obtained from a more detailed comparison of its structure with the crystal structures of full-length NagR and NagR-DBDs in complex with DNA [11]. In both latter complexes, DBD binding to palindromic DNA goes hand in hand with a non-canonical distortion of the B-DNA conformation. However, a rigid-body transformation of the ligand-free DasR structure onto the structure of NagR-DBDs in complex with DNA, which aligns the DBDs in the two structures, causes multiple steric clashes between helix α_E_6 from one DasR-DBD monomer and the NagR-DBD-bound DNA (Fig 7C). Thus, binding of DasR to DNA with ‘upwards’-oriented DBDs is sterically impeded when assuming that DasR binding to operator DNA induces a similar conformational distortion in the DNA than NagR.

**Fig 8:**
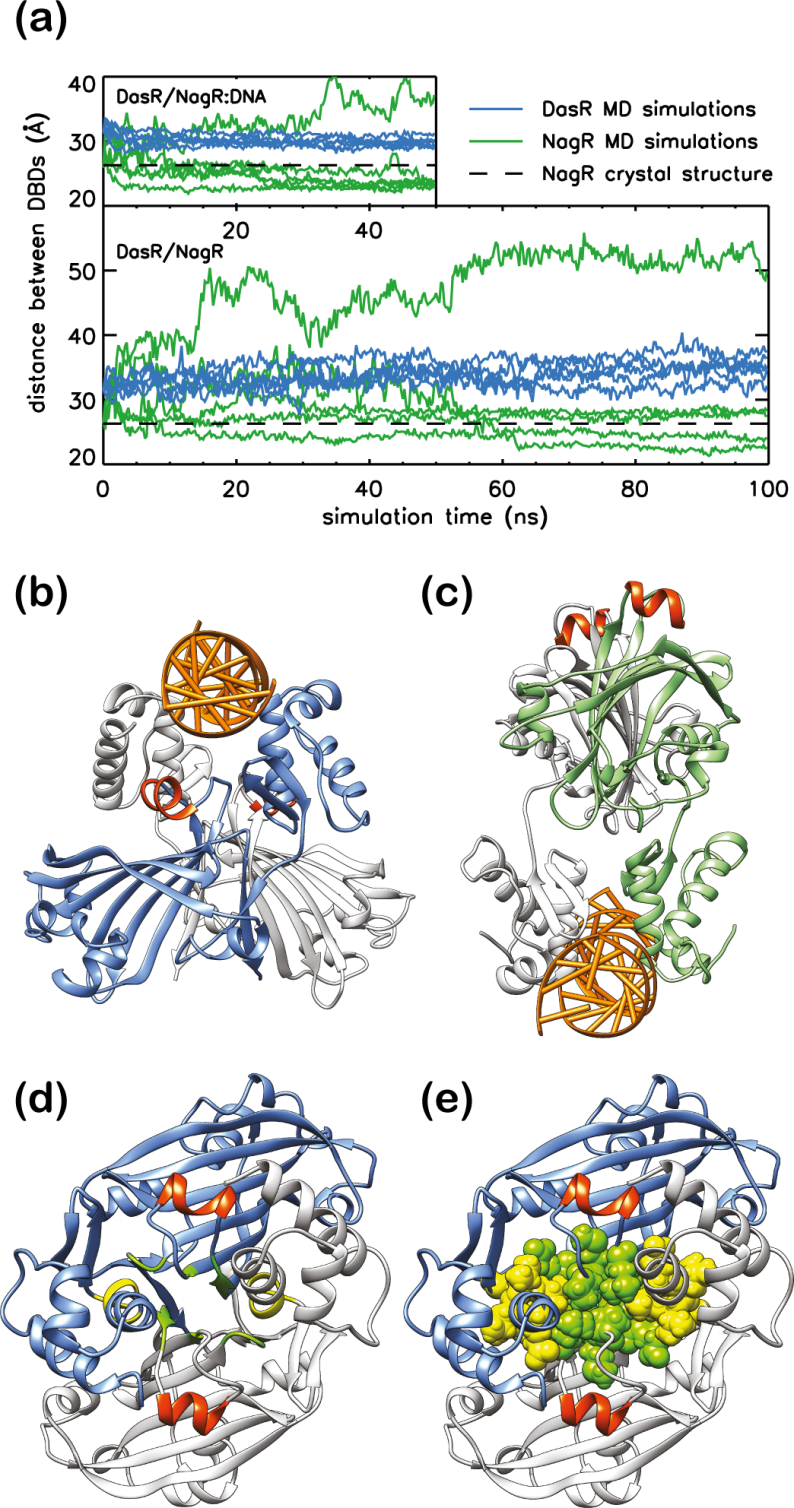
Impeded rapprochement of the DBDs in DasR. (a) DBD-COM distance of DasR (blue) and NagR (green) during MD simulations. While the upper plot displays the MD simulations performed in the presence of DNA, the lower plot shows the results from MD simulations in the absence of any DNA. The black dashed lines indicate the DBD-COM distance in the *dre*-site bound NagR crystal structure. Comparison of (b) a simulation snapshot of DasR bound to DNA and (c) the NagR-DNA complex crystal structure. DasR monomers are shown in blue and light grey, NagR monomers in green and light grey. Helices α_E_6 are coloured in orange red. (d) and (e) DasR in the ‘upwards’-directed conformation after 50 ns simulation time. Residues Thr197, Ser198, Leu199, Pro215, Met216, Gly240 and Asp241 are coloured in green, while residues Glu67, Leu68, Val69, Val70 and Glu71 are highlighted in yellow. In (e), the interaction-patch-forming residues are shown as spheres for further clarity.

In order to investigate the possibility that conformational rearrangements of the DasR-DBDs in the ‘upwards’ orientation enable binding to canonical B-DNA, the initial steps in the binding process of full-length DasR with ‘upwards’-oriented DBDs were addressed in more detail using atomistic molecular dynamics (MD) simulations. The behaviour of DasR during the simulation was compared to that of NagR with ‘downwards’-oriented DBDs. Additional simulations were performed to address the flexibility of the respective DBD-EBD orientation for both isolated DasR (in ‘upwards’ conformation) and NagR repressors (‘downwards’ orientation) in the absence of any DNA in solution. The initial binding of both repressors with the ‘upwards’ *versus* ‘downwards’ orientation of the DBDs to a *dre*-site-containing 30mer dsDNA fragment was investigated in six individual simulations. At the start of the simulations, the transcription regulators and the DNA segments were artificially separated by 11 Å in comparison to their positioning in the crystal structure of the NagR-DNA complex thus focussing on the last steps in the approach of the repressors to DNA. During each simulation contacts between the DBDs and the DNA were formed, however the residue-specific binding interactions observed in the NagR-DNA-complex crystal structure could not be fully reproduced on the accessible time scale. The final structures display merely intermediates of the binding process since the simulation times were limited to 50 ns– 100 ns. Nevertheless, clear differences between NagR and DasR can be observed. Thus the centre of mass (COM) distance between the DNA-interacting NagR-DBDs, which is approximately 26 Å in the NagR-DNA-complex crystal structure, shows a wide range of values ranging from 22 Å to 40 Å in the simulations (Fig 8A). The DBD-COM distances in DasR, however, are restricted to values between 28 Å and 33 Å. Any further rapprochement of the DasR-DBDs appears to be sterically hindered, and a DNA-binding mode similar to that observed in NagR cannot be accomplished (Fig 8B and 8C). Yet, this hindrance does not involve helix α_E_6 since the DNA segment retains the canonical B-DNA conformation in all simulations. Similar to the DNA-bound case, the isolated repressors display a DBD-orientation-dependent flexibility: the ‘upwards’-oriented DBDs sample a narrow distance range between 30 and 40 Å as compared to 22 to 55 Å for the ‘downwards’-oriented DBDs of NagR (Fig 8A).

The MD simulations show that the flexibility of the ‘upwards’-oriented DBDs is severely restricted by van-der-Waals interactions and hydrogen bonds that attach the DBDs to the EBDs. Identical results are obtained when performing the MD simulations in presence or absence of DNA. During the course of the simulations, an extensive interaction patch is formed across the EBD-DBD interface of DasR that includes EBD residues from the C-terminus of β_E_4, the N-terminus of β_E_5 and from the loop region interconnecting β_E_6 and β_E_7 (Fig 8D and 8E) as well as DBD residues located towards the C-terminus of α_D_3. These extensive interactions between the DBDs and EBDs prevent the two DasR-DBDs from getting closer to each other. Furthermore, hydrogen bonds are formed between residues Val70 from α_D_3 and Arg242 from the C-terminal end of the EBD as well as various residues from the linker segment between DBD and EBD of the same monomer (Lys86, Pro87 and Lys88) (S3 Fig). Taken together, these data suggest that the crystal structure of ligand-free full-length DasR represents a DNA-binding incompetent conformation.

### Sequence alignments hint that key DNA- and effector-binding determinants might be retained in other GntR/HutC repressors

In order to identify structural features that might be conserved across different GntR/HutC family members, a multiple sequence alignment was generated for all members for which structural data are available at the protein data bank (S2 Table) [17]. Pairwise sequence identities among these repressors range from 15 to 40% (S3 Table). Although most deposited GntR/HutC structures lack structural information for the DBDs, the multiple sequence alignment reveals nonetheless strong sequence conservation among those residues that participate in DNA binding as observed in NagR (S4 Fig) [11]. Thus, a strict conservation or a replacement with an amino acid with similar physico-chemical properties is often observed for those residues that in the NagR-DNA complex interact non-specifically with the sugar-phosphate backbone of the DNA *via* their side chains (Tyr12, Ser47, Thr50, Arg52, Gln53 and Thr72). In contrast, residues that show main-chain-mediated DNA-backbone interactions (Ile11 and Glu37) are distinctly less well conserved. In NagR, a glycine residue (Gly69) has been identified that is important for anchoring the tip of the wing motif of the wHTH DBD domain into the minor groove of the DNA [11]. Except for TraR from *Streptomyces phaeochromogenes*, this glycine residue occurs at an equivalent position in all aligned GntR/HutC transcription factors (S4 Fig). These findings suggest that the previously described overall DNA-binding mode of NagR is expected to be largely retained in other GntR/HutC family regulators [11].

The operator sequences recognised by DasR (*dre*-sites) have been characterised in great detail [20]. Since many residues involved in DNA interactions are strictly conserved between DasR and NagR, it can be anticipated that the atomic determinants identified for the specific DNA recognition of NagR also hold true for the recognition of *dre*-sites by DasR. Thus, two consecutive guanines, that are recognised in NagR by arginine residues Arg38 from helix α_D_2 and Arg48 from α_D_3, are conserved throughout *dre*-sites [20]. However, when considering other GntR/HutC members then only the arginine corresponding to Arg48 in NagR is retained in more than half of the aligned sequences (S4 Fig). Therefore, only one of the two guanines might be present at an equivalent position in the operator-binding sites of other GntR/HutC repressors, which is in agreement with the expectation that different repressors recognise different operator sequences.

**Fig 9:**
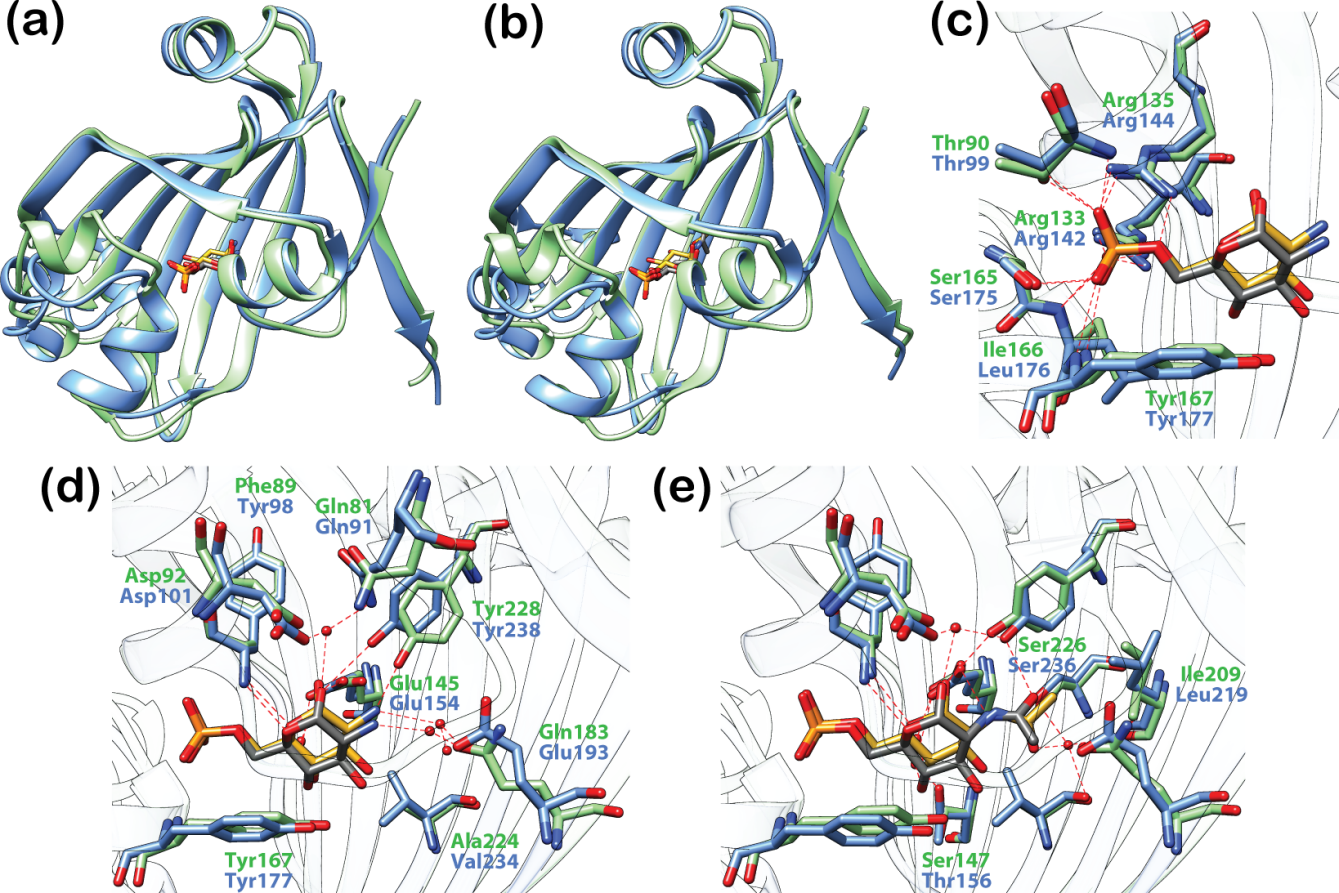
Effector recognition is highly similar in DasR and NagR. (a) and (b) Superposition of DasR-EBD (blue) and NagR (green; truncated to NagR-EBD) in complex with the phosphorylated sugars GlcN-6-P (a) and GlcNAc-6-P (b). The r.m.s. deviations of the Cα-positions of the superimposed structures are (a) 1.39 Å (a) and (b) 1.47 Å. For clarity, only the protein chain that is coordinating the α-anomer of GlcN-6-P or GlcNAc-6-P in the dimeric repressor is displayed. The phosphorylated sugars are depicted as stick models and coloured in grey (bound by DasR) or gold (bound by NagR). (c) Interaction of the phosphate moiety of GlcN-6-P with DasR and NagR. (d) and (e) Interaction of the sugar moiety of GlcN-6-P (d) and GlcNAc-6-P (e) with DasR and NagR. Protein residues are presented as stick models. Water molecules are shown as red spheres.

DasR and NagR share a highly similar GlcN-6-P- and GlcNAc-6-P-interaction pattern (Fig 9). The phosphate-moiety-coordinating arginine residues displayed from β-strand β_E_2 in DasR (Arg142 and Arg144) and NagR (Arg133 and Arg135) are largely conserved among other GntR/HutC repressors. This is reflected by a widely shared Arg-[Val/Leu/Ile]-Arg motif (S4 Fig). Moreover, in those repressors in which this motif is incomplete or absent, alternative arginine residues from distant sequence segments are displayed close in space in the 3D structures. These residues might therefore be able to substitute for any missing arginines in strand β_E_2. For example, SauR from *Streptomyces avermitilis* misses the aforementioned arginines, but Arg86 and Arg95 appear to be positioned at adequate distances in order to be able to interact with a putative phosphorylated effector. The same applies to PhnF from *Mycobacterium smegmatis* where it was recently revealed that Arg98 interacts with a sulphate molecule in the effector-binding pocket and thereby compensates for the missing arginine at position 131 [18]. Hence, the reoccurrence of arginine residues in the effector-binding site of GntR/HutC family members could indicate that many of these repressors preferentially interact with negatively charged effectors.

Additional observations suggest that these charged effectors might correspond to phosphorylated sugar molecules. For example, the observed binding of GlcN-6-P and GlcNAc-6-P to the N-termini of helices α_E_1 and α_E_5 in NagR and DasR is typical for the binding of nucleotides or dinucleotides to proteins, or the interaction of proteins with the sugar-phosphate backbone of DNA [27–29]. Furthermore, many residues that directly interact with the sugar moiety of GlcN-6-P and GlcNAc-6-P in NagR and DasR are also conserved in other GntR/HutC family members. Accordingly, Glu154 in DasR, which forms a hydrogen bond with the hydroxyl group at position C1 of the phosphorylated sugars (Fig 9D and 9E), is either strictly conserved or at least replaced by a similar amino acid in two-thirds of the aligned sequences (S4 Fig). In all sequences, except for SauR, an aromatic amino acid is present at a position equivalent to that of Tyr177, whose side chain stacks against the sugar ligand in DasR. Partially conserved additional residues are Arg221, which is located ‘below’ the sugar moiety and is involved in hydrophobic interactions, as well as Tyr238, which is located ‘above’ the sugar moiety and directly interacts with the hydroxyl group at C1 of GlcN-6-P (Fig 2A) or the carboxyl group in the acetyl moiety of GlcNAc-6-P (Fig 2B). These observations indicate that although the nature of the effector molecules is currently unknown for many regulators displayed in the multiple sequence alignment, it appears quite possible that in this class of prokaryotic transcription factors negatively charged carbohydrates might play a prominent role as allosteric effectors.

## Discussion

### DasR and NagR share numerous effector-binding features

A structural superposition of effector-bound DasR-EBD and previously described NagR reveals close similarities between the EBDs of the two transcription factors in the effector-bound states (Fig 9A and 9B). The two repressors, which share an overall sequence identity of 38.8% (S3 Table) and a sequence identity of 34.8%, when considering the EBDs, only, display highly similar interactions with the phosphorylated sugars. More precisely, both DasR and NagR coordinate the phosphate moiety of the effectors *via* the N-termini of helices α_E_1 and α_E_5, and *via* two arginine residues from β-strand β_E_2 (Fig 9C) As anticipated from the high sequence similarity between the two repressor molecules, the sugar moieties of GlcN-6-P and GlcNAc-6-P bear also a high resemblance in their interactions with the effector-binding pockets in the two transcription factors (Fig 9D and 9E; S5 Fig). However, while NagR binds the small molecule effectors exclusively in an α-anomeric configuration [11], DasR-EBD shows a selective binding of the ligand α-anomer to one binding site and of the β-anomer to the second site. It remains currently unclear whether this reflects a DasR-specific cooperative ligand-binding mechanism or whether this behaviour is caused by either crystal packing asymmetries or by the truncation of the protein chain in the DasR-EBD variant.

When extending the comparison of DasR and NagR to other GntR/HutC family regulators then the sequence conservations in both the DBDs and the EBDs of these repressors suggest that not only the overall effector and DNA-binding modes are conserved in this family but that also negatively charged sugar molecules should be preferentially tested as putative effector molecules for the orphan bacterial repressor molecules from this family.

### Common aspects of the allosteric regulation of GntR/HutC repressors

Members of the GntR/HutC family constitute so-called one-component signal transduction elements and in the case of DasR and NagR link environmental stimuli to cellular responses [3]. In order to understand the allosteric regulation of these transcription factors, insight has to be gained into the atomic coupling mechanism that allows for the modulation of the DBD-mediated DNA-binding affinities of the repressors upon binding of small molecular effectors to the sterically distant EBDs.

In DasR, NagR and many additional GntR/HutC repressors the fold of the EBD appears to be considerably more flexible in the absence of any EBD-bound ligand. This heightened flexibility is not only reflected by difficulties in modelling certain EBD segments in these ligand-free crystal structures but also by the observation that the full-length proteins appear to be prone to unintentional proteolytic cleavage during crystallisation. Thus, attempts to crystallise full-length GntR/HutC repressors in the absence of effectors often resulted in crystals containing only the dimeric EBDs (S2 Table). At the same time numerous segments of the EBDs, which most frequently comprise helix α_E_1 and adjacent segments, can often not be modelled in these structures. This also extends to the linker segment between DBD and EBD, which adopts different conformations in ligand-free DasR and DasR-EBD (see above). This heightened flexibility can also be observed in the structure of DNA-bound full-length NagR. Here too, a number of segments in the effector-free EBD could not be modelled [11].

Conversely, a comparison of effector-bound structures shows that effector binding causes an overall stabilisation of the EBDs. Upon effector binding all EBD segments are clearly defined in the electron density maps of all effector-bound structures determined so far. This holds particularly true for helix α_E_1 and adjacent segments such as the interdomain linker segment that forms β-strand β* upon effector binding. Moreover, formation of β* is accompanied by an ‘upwards’ positioning of the DBDs.

The effector-induced ‘upwards’ positioning of the DBDs appears to impede DNA binding, since all currently available data suggest that the juxtaposed and concerted binding of two DBDs belonging to a single dimeric repressor can only occur with ‘downwards’-oriented DBDs. This was directly observed in the crystal structure of NagR in complex with DNA [11] and in the case of DasR investigated by MD simulations, as reported here. The data presented here suggest that effector-induced stabilisation of the EBD, formation of β-strand β* and the concomitant ‘upwards’ positioning of the DBDs are responsible for abolishing DNA binding and are therefore key to the allosteric coupling mechanism that characterises the GntR/HutC family members.

### Towards a unified model for the allosteric regulation of GntR/HutC repressors

Although it appears tempting to propose that the allosteric regulation of GntR/HutC repressors is comprehensibly characterised by a simple two-state allosteric model that toggles between ‘upwards’- and ‘downwards’-oriented DBDs, such a model would not give justice to the full range of structural information available for GntR/HutC repressors. A first indication for this comes from the observation that in full-length NagR not only two but three distinct overall conformations have been observed. In addition to the DNA-bound and effector-bound conformation, yet another orientation of the DBDs is adopted in sulphate-bound NagR [11, 16]. When comparing the structures of different GntR/HutC repressors then it becomes obvious that the positioning of the DBDs is extremely diverse in these structures (Fig 6). This also holds true for DasR and NagR, which share high sequence homology. Although the DBDs of both DasR and NagR are oriented in an ‘upwards’ position upon formation of β-strand β*, the exact orientation of the DBDs still significantly differs between these two repressors. This also applies to other GntR/HutC repressors displaying β-strand β* (S6 Fig).

**Fig 10:**
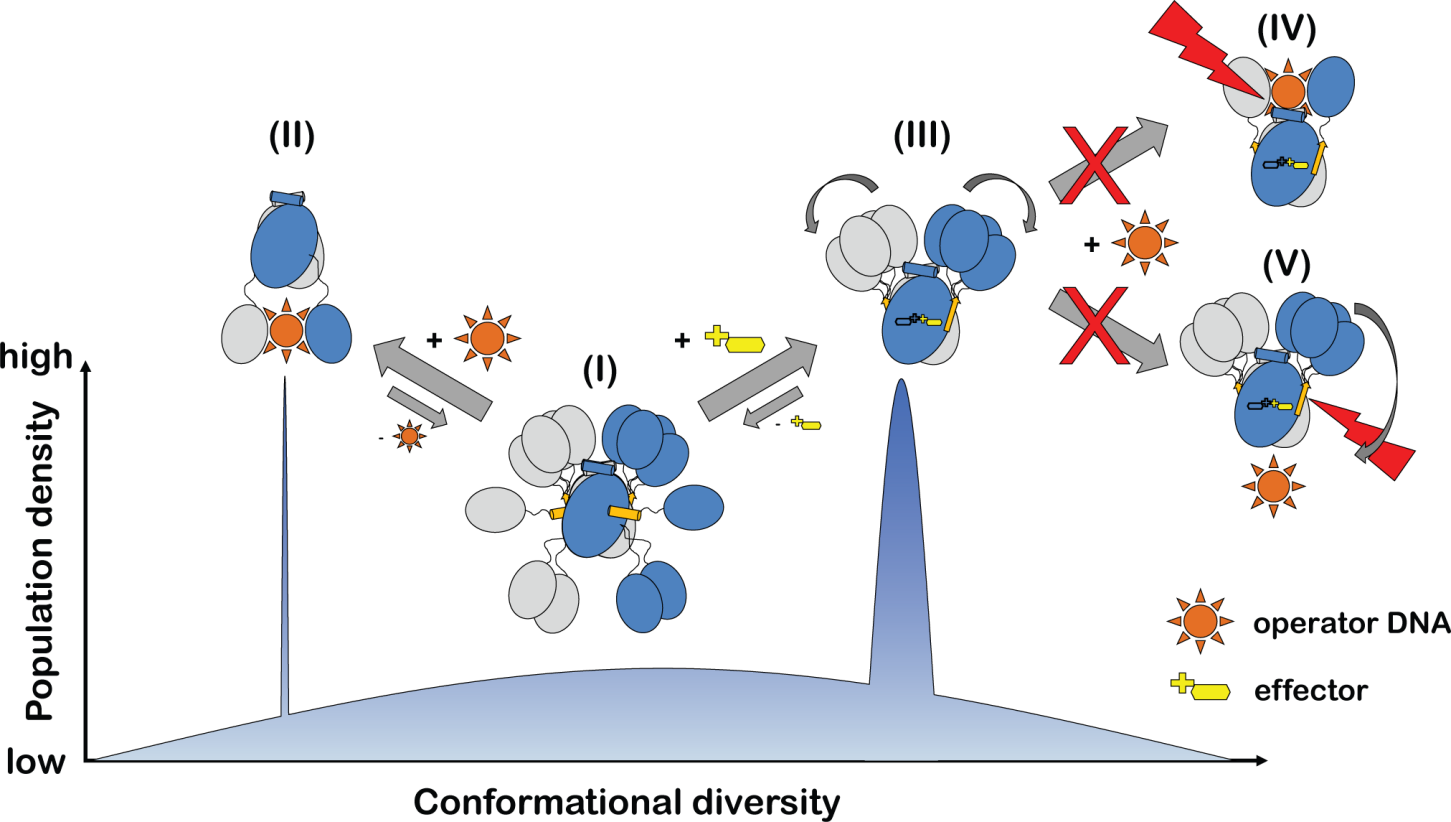
A conformational selection model best describes the allosteric regulation of GntR/HutC transcription factors. The population density of distinct functional states is schematically compared to the conformational diversity observed in GntR/HutC crystal structures. The different functional states are (I) effector- and DNA-free repressor, (II) DNA-bound repressor and (III) effector-bound repressor. Effector-bound repressors cannot bind DNA in the conformations depicted in (IV) and (V).

These observations can, however, be reconciled when switching from a two-state allosteric model to a conformational selection model (Fig 10) [30, 31]. Thus in the case of the GntR/HutC repressors, it appeared that in the absence of any effector the DBDs are only loosely attached to the EBDs and the flexibility of the linker segment allows the DBDs to sample a great variety of different orientations. Among these are conformations with the DBDs oriented in both the ‘upwards’ (as described above for ligand-free NagR) and the ‘downwards’ position. Conversely, effector binding shifts the distribution of the different conformations towards the ‘upwards’ conformation. On an atomic level, effector binding goes hand in hand with distinct rearrangements in the effector-binding domain resulting in the repositioning of helices α_E_1 and α_E_5, and the formation of β-strand β*. Interestingly, however, this does not necessarily have to lock the DBDs into a fixed orientation, but the movement of the DBDs is now restricted to the upper part of the effector-binding domain. A displacement of the DBDs towards the ‘downwards’ position that would be required for the juxtaposed binding of the DBDs to the operator DNA sequence is impeded once β* is formed (Fig 10).

We previously proposed that the allosteric regulation of GntR/HutC repressors could be described by a ‘jumping jack’-like movement of the DBDs. However, the currently available data on GntR/HutC repressors indicate that the ‘jumping jack’ model might overstate the importance of individual conformational states. Instead, allosteric regulation in GntR/HutC repressors might better be described by a conformational selection model in which the main function of the effector is to lock β-strand β* into place, thereby holding the DBDs ‘on a shorter leash’ and as a consequence impeding DNA binding. Further mutational analyses, such as the lengthening and shortening of the linker segment between the EBD and the DBD will be helpful to further ascertain the validity of this mechanism.

The question as to what extent allosteric regulation is caused primarily by an effector-induced toggling between defined conformational states or by alterations in the dynamic behaviour of the protein in question remains under constant debate in structural biology [32]. Moreover, it is often difficult to assess the relative contribution of any enthalpic (defined conformational states) versus entropic (changes in the dynamic behaviour) effects to the allosteric regulation of a protein since often different experimental approaches are required for studying those. In case of the well-studied bacterial repressor TetR, for example, folding and limited proteolysis studies highlighted the importance of effector-induced changes in the dynamic behaviour of the protein for its allosteric regulation, whereas manifold structural studies emphasized the importance of defined conformational states [33–35]. In this regard the GntR/HutC repressors might be of interest for further studies since here crystallographic structure determinations were able to shed light on an interplay between changes in the dynamic behaviour, namely on the effector-induced stabilisation of the EBD and the direct enthalpic consequences, namely the formation of a tighter grip on the leash that holds the DBDs.

## Materials and Methods

### Protein production and purification

DasR and DasR-EBD were overexpressed in E. *coli* BL21 (DE3) pREP4::*groESL* cells (Novagen, EMD Biosciences, Darmstadt, Germany) utilizing pET15b vectors (Novagen, EMD Biosciences, Darmstadt, Germany) that contain either the *dasR* (residues 1–254; UniProtKB O34817, [19]) or the *dasR-ebd* gene (residues 88–254). Both constructs display an additional N-terminal hexahistidine tag and a thrombin cleavage site. The cells were grown at 310 K in LB medium containing 100 mg/mL ampicillin and 10 mg/mL kanamycin as selection markers. At an OD_600_ of 0.6, the temperature was decreased to 293 K and protein expression was induced by adding 0.5 mM isopropyl-*β*-*D*-thiogalactopyranoside (IPTG). About two hours after reaching the stationary growth phase, the cells were harvested by centrifugation and the respective cell pellet was stored at 193 K. Prior to protein purification of DasR or DasR-EBD, the respective cells were thawed and suspended in a buffer containing 50 mM sodium phosphate pH 8.0, 300 mM NaCl and 20 mM imidazole (10 mL buffer per g cells). Consecutively, 1 mM phenylmethanesulphonyl fluoride (PMSF), 1 mg/mL lysozyme and 1 μg/mL DNaseI were added to assist cell lysis, followed by cell disruption *via* sonication. The sample was subsequently centrifuged for 1 h at 95,000 g and 277 K. After filtration through a 0.45 μm filter, the supernatant was loaded onto a 10 mL Ni^2+^-bound HisTrap FF crude column (GE Healthcare, Munich, Germany). Both DasR and DasR-EBD were eluted *via* a linear gradient of 0–500 mM imidazole in 50 mM sodium phosphate buffer pH 8.0 supplemented with 300 mM NaCl. Fractions containing the target protein were identified *via* SDS-PAGE [36] and pooled. The N-terminal hexahistidine tag was cleaved off overnight at 291 K with thrombin (40 NIH units per mg DasR and 5 NIH units per mg DasR-EBD).

In the case of DasR-EBD, an additional purification step was conducted to effectively separate the target protein from thrombin. For that purpose, the sample was diluted tenfold with 20 mM Tris-HCl buffer pH 7.5 containing 50 mM NaCl and applied to a 20 mL Q Sepharose Fast Flow anion exchange column (GE Healthcare, Munich, Germany). The sample was eluted *via* a linear gradient of 0–1 M NaCl in 20 mM Tris-HCl buffer pH 7.5.

In a final chromatographic step, full-length DasR was applied to a Hi-Load 26/60 Superdex 200 column (GE Healthcare, Munich, Germany) pre-equilibrated with 30 mM sodium phosphate buffer pH 8.0 containing 200 mM NaCl, while DasR-EBD was applied to a HiLoad 26/60 Superdex 75 column (GE Healthcare, Munich, Germany) equilibrated with 20 mM Tris-HCl buffer pH 7.5 supplemented with 150 mM NaCl. Elution fractions containing the highly pure target protein were identified *via* SDS-PAGE, pooled and concentrated with Vivaspin centrifugal filter devices (Vivascience, Hannover, Germany). Until further usage, 50 μL aliquots were flash-frozen in liquid nitrogen and stored at 193 K.

### Protein crystallisation

Diffraction quality crystals of ligand-free DasR were obtained *via* the sitting-drop vapour-diffusion method by mixing 0.2 μL DasR (7.9 mg/mL DasR in 30 mM sodium phosphate pH 8.0, 200 mM NaCl) with 0.4 μL reservoir solution (0.1 M sodium citrate tribasic dihydrate pH 5.5, 26% (w/v) polyethylene glycol (PEG) 400, 10% (v/v) isopropanol) and equilibrating the droplets against 50 μL reservoir solution at 292 K. Crystals were flash-cooled in liquid nitrogen with 20% (v/v) ethylene glycol as a cryoprotectant.

Crystallisation of ligand-free DasR-EBD was achieved *via* the sitting-drop vapour-diffusion method by combining 0.2 μL DasR-EBD (5.6 mg/mL DasR in 20 mM Tris-HCl pH 7.5, 150 mM NaCl) and 0.2 μL reservoir solution consisting of 0.1 M Bis-Tris propane pH 9.0, 4.0 M potassium formate and 20% (w/v) PEG monomethyl ether 2,000. The droplets were equilibrated against 50 μL reservoir solution at 292 K. Diffraction quality crystals were flash-cooled in liquid nitrogen using 20% (v/v) ethylene glycol as a cryoprotectant.

DasR-EBD in complex with GlcN-6-P and GlcNAc-6-P was crystallised using the sitting-drop vapour-diffusion method by mixing 0.4 μL DasR-EBD (5.6 mg/mL DasR-EBD in 20 mM Tris-HCl pH 7.5, 150 mM NaCl containing 10 mM GlcN-6-P or GlcNAc-6-P) with 0.2 μL reservoir solution and equilibrating the droplets against 50 μL reservoir solution at 292 K. Diffraction quality crystals were obtained after several days with a reservoir solution consisting of 0.1 M potassium thiocyanate and 30% (w/v) PEG monomethyl ether 2,000. The crystals were flash-frozen in liquid nitrogen using 20% (v/v) ethylene glycol as cryoprotectant.

### Diffraction data collection, structure determination and refinement

Diffraction data sets of ligand-free DasR and DasR-EBD as well as of DasR-EBD in complex with GlcN-6-P or GlcNAc-6-P were collected from single crystals at 100 K at synchrotron beamline BL14.1 operated by the Helmholtz-Zentrum Berlin (HZB) at the BESSY II electron storage ring (Berlin-Adlershof, Germany [37]). Data were indexed and integrated with XDS and scaled with XSCALE [38, 39]. Initial phases for each data set were determined *via* molecular replacement with PHASER [40] using the isolated DNA- and effector-binding domain of the homologous transcriptional regulator NagR from *B*. *subtilis* (PDB-ID 2WV0, [16]) as search models. Molecular replacement solutions were readily obtained, and the resulting models stepwise completed by multiple cycles of manual model building with COOT [41] and automated refinement with PHENIX [42]. The α-anomers of the molecules GlcN-6-P and GlcNAc-6-P were loaded *via* the LIBCHECK plug-in of COOT using their 3-letter codes GLP and 16G, respectively and were placed into unambiguous residual electron density. In addition, the pdb-files of the corresponding β-anomers located in one monomer of each effector-bound DasR-EBD dimer and the associated geometry parameters were generated with JLIGAND [43]. The quality of the final models was validated with MOLPROBITY [44]. Crystallographic data collection and refinement statistics are summarised in Table 1. All structural illustrations were prepared with CHIMERA [45]. The atomic models have been deposited with the protein data bank and can be accessed with IDs: 4ZSI, 4ZSK, 4ZSB and 4ZS8.

### Structure analysis

Protein-ligand interactions were analysed using LIGPLOT+ [46]. For comparisons, the structures were superimposed with LSQKAB [47] from the CCP4 Software Suite [48]. The identification and calculation of pocket volumes in the crystal structures of ligand-free DasR-EBD, ligand-free DasR and effector-bound DasR-EBD was performed *via* the CASTp server using a solvent probe radius of 1.4 Å [49]. For ligand-bound DasR-EBD, the calculation was carried out after omission of the small molecule effectors from the binding pockets. Pocket volumes in ligand-free DasR-EBD, which forms a biological homodimer *via* a crystallographic 2-fold rotational axis, were calculated for the whole dimer and subsequently divided in half for chain-specific comparisons.

### Molecular dynamics simulations

For the MD simulations of spontaneous DNA binding, a periodic 30mer dsDNA fragment was designed containing the palindromic *dre*-site sequence [11]. Thereby, possible interactions with charged termini of the DNA were avoided. The structure of full-length *dre*-site-bound NagR (PDB-ID 4WWC) was fitted to the corresponding sequence on the 30mer dsDNA fragment by superimposing the sugar-phosphate backbone. Structural gaps in the original crystal structure were filled *via* model building using COOT, while missing side chain atoms were added using MODELLER [50]. Prior to starting the simulations, NagR was translated by 11 Å away from the DNA. For comparison to NagR, the ligand-free DasR crystal structure was fitted on NagR by superimposing the DasR-DBDs onto the NagR-DBDs. Thus, the DBDs of both proteins were facing the *dre*-site element in the same orientation, resulting in equivalent starting positions for NagR and DasR. The chosen rectangular periodic box had the dimensions of 104 Å x 104 Å x 100 Å. Finally, the systems were solvated using the TIP3P water model [51]. Na^+^ and Cl^-^ ions were added at a physiological ion concentration of 0.15 M. The systems comprised more than 35,000 water molecules. For comparison, additional systems of the isolated NagR and DasR proteins in solution (0.15 M NaCl) were set up using a rhombic dodecahedron box with a minimal separation of 2 nm between the protein and the box. The systems comprised more than 35,000 water molecules.

The MD simulations were carried out using GROMACS 4.6 [52]. The Amber ff99SB-ILDN force field was used for protein, ion and DNA [53, 54]. The bsc0-refinement was applied to keep the DNA backbone stable [55]. The systems were minimised for 200 steps using the steepest descent algorithm, followed by a 1 ns NVT equilibration with position restraints on all heavy atoms (200 ps for isolated repressor systems). For both DNA-containing systems (DasR and NagR), six production simulations of 50 ns length each were performed in the NpT ensemble. The isolated repressors were studied in five simulations, each (100 ns).

One simulation of each DNA-repressor system was extended to 100 ns. The temperature of 300 K (310 K for isolated repressors) was kept constant applying the velocity rescale thermostat with a time constant of 0.1 ps [56]. The pressure was set to 1 bar and controlled by a Parinello-Rahman barostat with a time constant of 2 ps [57]. In order to prevent the infinite DNA fragment from being distorted, a semi-isotropic pressure coupling with a compressibility of 4.5 x 10–5 bar^-1^ was applied, using a fixed box size in z-direction (isotropic pressure coupling for isolated repressor systems). Pauli repulsion and van-der-Waals interactions were described by a Lennard-Jones potential with a short-range cut-off of 1.4 nm. Electrostatic interactions were treated using a cut-off distance of 0.9 nm for short-ranged interactions and the PME method for long-range interactions beyond the cut-off [58]. An integration time step of 2 fs was used.

## Supporting Information

S1 Fig Closeup view of the effector-binding site of DasR-EBD. The stereo views show the interaction of DasR-EBD with the β-anomeric configuration of (a) GlcN-6-P and (b) GlcNAc-6-P. GlcN-6-P, GlcNAc-6-P and the interacting protein residues are presented as stick models and water molecules are depicted as red spheres.
(PDF)

S2 Fig Electron density maps of the effector-binding site of DasR-EBD with GlcN-6-P and GlcNAc-6-P. Simulated annealing *F*_*o*_*-F*_*c*_ OMIT maps show the GlcN-6-P-binding site of (a) chain A and (b) chain B as well as the GlcNAc-6-P-binding site of (c) chain A and (d) chain B in the respective effector-bound structures of DasR-EBD. Maps were calculated with PHENIX and are contoured as green mesh at 3.0 σ. Sugar molecules and all protein residues omitted during refinement and map calculation are shown as stick models. Water molecules are presented as red spheres. (e) and (f) Magnification of GlcNAc-6-P in (e) chain A and (f) chain B showing the corresponding simulated annealing *F*_*o*_*-F*_*c*_ OMIT maps to illustrate the unambiguity of the ligand position and its anomeric configuration.
(PDF)

S3 Fig Distinct hydrogen bonds keep the DBDs of DasR in an ‘upwards’ position. A snapshot from a simulation (a) with a corresponding zoomed view (c), as well as the crystal structure (b) with a corresponding zoomed view (d) is shown. Selected regions in (a) and (c) were coloured as in Fig 8. Specific residues are illustrated as stick model. Residue Glu196 originates from the neighbouring chain of the biological dimer.
(PDF)

S4 Fig Sequence alignment of structurally characterized members of the GntR/HutC transcription factor family. The sequence alignment was performed with CLUSTAL OMEGA [59] using the canonical protein sequences of the structurally characterized (full or partial) GntR/HutC family members specified in Supplemental S2 Table. Secondary structure elements refer to the topology of DasR and are marked with (h) or (s) for α-helices and β-strands, respectively. For a detailed classification, the familiar nomenclature α_D/E_ and β_D/E_ is used. Residues involved in DNA binding in NagR [11] and effector binding in DasR or NagR are highlighted by a coloured background. Residues involved in of DNA and effector binding, e.g. those forming base-specific contacts with the DNA, or hydrogen bonds as well as hydrophobic and CH/π interactions with the phosphorylated sugar, are additionally marked by a black arrow.
(PDF)

S5 Fig Sequence alignment of DasR-EBD from *S*. *coelicolor* and NagR-EBD from *B*. *subtilis*. The sequence alignment was performed with CLUSTAL OMEGA [59] using the canonical protein sequences of entries Q9K492 and O34817 from the UniProt database [19]. Secondary structure elements refer to the topology of DasR and are marked with (h) or (s) for α-helices and β-strands, respectively. For a detailed classification, the familiar nomenclature α_E_ and β_E_ is used. Residues of DasR or NagR involved in effector binding (as identified with LIGPLOT+ [46]) are highlighted by a blue-coloured background. If these amino acids are fully conserved in both transcription factors, they are additionally displayed in bold red letters. In general, fully conserved residues in both sequences are marked with an asterisk (*), while the conservation between groups of strongly and weakly similar properties is labelled with a colon (:) and a period (.), respectively.
(PDF)

S6 Fig Remaining DBD flexibility among GntR/HutC transcription factors with β-strand β* formed in the linker segment. (a) and (b) Superposition of the crystal structures of ligand-free SauR from *S*. *avermitilis* (grey, PDB-ID 3EET), ligand-free DasR from *S*. *coelicolor* (blue, PDB-ID 4ZS8), GlcNAc-6-P-bound NagR from *B*. *subtilis* (green, PDB-ID 4U0W) and ligand-free PhnF from *M*. *smegmatis* ((khaki, PDB-ID 3F8M) displayed as a cartoon representation in a side view (a) and a top view (b). The ligand GlcNAc-6-P bound to NagR is shown as a stick model. The EBDs of all four dimeric structures were rendered transparent for clarity.
(PDF)

S1 Table Comparison of the effector-binding sites (pockets) from the crystal structures of ligand-free DasR-EBD, ligand-free DasR and effector-bound DasR-EBD. The pocket volumes were calculated as described in the ‘Materials and Methods’ section.
(PDF)

S2 Table Structurally characterized members of the GntR/HutC transcription factor family. The listed regulators were identified by a protein structure database search (Dali Lite v.3, [60]) *via* the Dali server using the crystal structure of full-length DasR (PDB-ID 4ZS8) as a search model. From the resulting structures only those containing a GntR-family-specific wHTH domain as well as a HutC-subfamily-specific UTRA domain (as described in the respective entry in the UniProt database [19]) were used for a subsequent multiple sequence alignment *via* CLUSTAL OMEGA [59] that is shown in S4 Fig. For a better discrimination, regulators without an individual gene or protein name and mostly of unknown function were given unambiguous acronyms, e.g. ScuR for *S*. *c**oelicolor* unnamed Regulator. The panel “Match of characteristic residues” describes residues that are explicitly involved in DNA binding of NagR [11] and in effector binding of DasR and/or NagR (Fig 9), and their equivalents from other GntR/HutC family members inferred by structural comparison with DasR and NagR. Residues marked in red were only inferred by sequence due to the lack of structural information.
(PDF)

S3 Table Pairwise comparison of protein sequence identities between all GntR/HutC transcription factors used in the multiple sequence alignment shown in S4 Fig. The sequence identities were calculated with CLUSTAL OMEGA [59].
(PDF)

## References

[1] Jacob F, Monod J. Genetic regulatory mechanisms in the synthesis of proteins. J Mol Biol. 1961;3:318–356. . 13718526 10.1016/s0022-2836(61)80072-7

[2] Balleza E, Lopez-Bojorquez LN, Martinez-Antonio A, Resendis-Antonio O, Lozada-Chavez I, Balderas-Martinez YI, et al Regulation by transcription factors in bacteria: beyond description. FEMS Microbiol Rev. 2009;33(1):133–151. 10.1111/j.1574-6976.2008.00145.x . 19076632 PMC2704942

[3] Ulrich LE, Koonin EV, Zhulin IB. One-component systems dominate signal transduction in prokaryotes. Trends Microbiol. 2005;13(2):52–56. 10.1016/j.tim.2004.12.006 . 15680762 PMC2756188

[4] Haydon DJ, Guest JR. A new family of bacterial regulatory proteins. FEMS Microbiol Lett. 1991;63(2–3):291–295. . 2060763 10.1016/0378-1097(91)90101-f

[5] Finn RD, Bateman A, Clements J, Coggill P, Eberhardt RY, Eddy SR, et al Pfam: the protein families database. Nucleic Acids Res. 2014;42(Database issue):D222–230. 10.1093/nar/gkt1223 . 24288371 PMC3965110

[6] Hoskisson PA, Rigali S. Chapter 1: Variation in form and function the helix-turn-helix regulators of the GntR superfamily. Adv Appl Microbiol. 2009;69:1–22. 10.1016/S0065-2164(09)69001-8 . 19729089

[7] Rigali S, Derouaux A, Giannotta F, Dusart J. Subdivision of the helix-turn-helix GntR family of bacterial regulators in the FadR, HutC, MocR, and YtrA subfamilies. J Biol Chem. 2002;277(15):12507–12515. 10.1074/jbc.M110968200 . 11756427

[8] Jain D, Nair DT. Spacing between core recognition motifs determines relative orientation of AraR monomers on bipartite operators. Nucleic Acids Res. 2013;41(1):639–647. 10.1093/nar/gks962 23109551 PMC3592433

[9] van Aalten DM, DiRusso CC, Knudsen J. The structural basis of acyl coenzyme A-dependent regulation of the transcription factor FadR. EMBO J. 2001;20(8):2041–2050. Epub 2001/04/11. 10.1093/emboj/20.8.2041 . 11296236 PMC125426

[10] Xu Y, Heath RJ, Li Z, Rock CO, White SW. The FadR.DNA complex. Transcriptional control of fatty acid metabolism in Escherichia coli. J Biol Chem. 2001;276(20):17373–17379. Epub 2001/03/30. 10.1074/jbc.M100195200 . 11279025

[11] Fillenberg SB, Grau FC, Seidel G, Muller YA. Structural insight into operator dre-sites recognition and effector binding in the GntR/HutC transcription regulator NagR. Nucleic Acids Res. 2015;43(2):1283–1296. 10.1093/nar/gku1374 . 25564531 PMC4333415

[12] Hong M, Fuangthong M, Helmann JD, Brennan RG. Structure of an OhrR-ohrA operator complex reveals the DNA binding mechanism of the MarR family. Mol Cell. 2005;20(1):131–141. 10.1016/j.molcel.2005.09.013 . 16209951

[13] Allison SL, Phillips AT. Nucleotide sequence of the gene encoding the repressor for the histidine utilization genes of Pseudomonas putida. J Bacteriol. 1990;172(9):5470–5476. . 2203753 10.1128/jb.172.9.5470-5476.1990 PMC213214

[14] Aravind L, Anantharaman V. HutC/FarR-like bacterial transcription factors of the GntR family contain a small molecule-binding domain of the chorismate lyase fold. FEMS Microbiol Lett. 2003;222(1):17–23. . 12757941 10.1016/S0378-1097(03)00242-8

[15] Gorelik M, Lunin VV, Skarina T, Savchenko A. Structural characterization of GntR/HutC family signaling domain. Protein Sci. 2006;15(6):1506–1511. 10.1110/ps.062146906 . 16672238 PMC2242532

[16] Resch M, Schiltz E, Titgemeyer F, Muller YA. Insight into the induction mechanism of the GntR/HutC bacterial transcription regulator YvoA. Nucleic Acids Res. 2010;38(7):2485–2497. 10.1093/nar/gkp1191 . 20047956 PMC2853113

[17] Berman HM, Westbrook J, Feng Z, Gilliland G, Bhat TN, Weissig H, et al The Protein Data Bank. Nucleic Acids Res. 2000;28(1):235–242. . 10592235 10.1093/nar/28.1.235 PMC102472

[18] Gebhard S, Busby JN, Fritz G, Moreland NJ, Cook GM, Lott JS, et al Crystal structure of PhnF, a GntR-family transcriptional regulator of phosphate transport in Mycobacterium smegmatis. J Bacteriol. 2014;196(19):3472–3481. 10.1128/JB.01965-14 . 25049090 PMC4187667

[19] UniProt-Consortium. Activities at the Universal Protein Resource (UniProt). Nucleic Acids Res. 2014;42(Database issue):D191–198. 10.1093/nar/gkt1140 . 24253303 PMC3965022

[20] Colson S, Stephan J, Hertrich T, Saito A, van Wezel GP, Titgemeyer F, et al Conserved cis-acting elements upstream of genes composing the chitinolytic system of streptomycetes are DasR-responsive elements. J Mol Microbiol Biotechnol. 2007;12(1–2):60–66. 10.1159/000096460 . 17183212

[21] Rigali S, Nothaft H, Noens EE, Schlicht M, Colson S, Muller M, et al The sugar phosphotransferase system of Streptomyces coelicolor is regulated by the GntR-family regulator DasR and links N-acetylglucosamine metabolism to the control of development. Mol Microbiol. 2006;61(5):1237–1251. 10.1111/j.1365-2958.2006.05319.x . 16925557

[22] Tenconi E, Urem M, Swiatek-Polatynska MA, Titgemeyer F, Muller YA, van Wezel GP, et al Multiple allosteric effectors control the affinity of DasR for its target sites. Biochem Biophys Res Commun. 2015;464(1):324–329. 10.1016/j.bbrc.2015.06.152 . 26123391

[23] Rigali S, Titgemeyer F, Barends S, Mulder S, Thomae AW, Hopwood DA, et al Feast or famine: the global regulator DasR links nutrient stress to antibiotic production by Streptomyces. EMBO Rep. 2008;9(7):670–675. 10.1038/embor.2008.83 . 18511939 PMC2475330

[24] Swiatek-Polatynska MA, Bucca G, Laing E, Gubbens J, Titgemeyer F, Smith CP, et al Genome-Wide Analysis of In Vivo Binding of the Master Regulator DasR in Streptomyces coelicolor Identifies Novel Non-Canonical Targets. PLoS One. 2015;10(4):e0122479 10.1371/journal.pone.0122479 . 25875084 PMC4398421

[25] Swiatek MA, Tenconi E, Rigali S, van Wezel GP. Functional analysis of the N-acetylglucosamine metabolic genes of Streptomyces coelicolor and role in control of development and antibiotic production. J Bacteriol. 2012;194(5):1136–1144. 10.1128/JB.06370-11 . 22194457 PMC3294797

[26] Denker K, Orlik F, Schiffler B, Benz R. Site-directed mutagenesis of the greasy slide aromatic residues within the LamB (maltoporin) channel of Escherichia coli: effect on ion and maltopentaose transport. J Mol Biol. 2005;352(3):534–550. 10.1016/j.jmb.2005.07.025 . 16095613

[27] Hol WGJ, Vanduijnen PT, Berendsen HJC. Alpha-Helix Dipole and Properties of Proteins. Nature. 1978;273(5662):443–446. 10.1038/273443a0 . 661956

[28] McCammon JA, Harvey SC. Dynamics of proteins and nucleic acids Cambridge Cambridgeshire; New York: Cambridge University Press; 1987 xii, 234 p. p.

[29] Murphy FV, Churchill MEA. Nonsequence-specific DNA recognition: a structural perspective. Structure with Folding & Design. 2000;8(4):R83–R89. 10.1016/S0969-2126(00)00126-X . 10801483

[30] Changeux JP. Allostery and the Monod-Wyman-Changeux model after 50 years. Annu Rev Biophys. 2012;41:103–133. 10.1146/annurev-biophys-050511-102222 . 22224598

[31] Gunasekaran K, Ma B, Nussinov R. Is allostery an intrinsic property of all dynamic proteins? Proteins. 2004;57(3):433–443. 10.1002/prot.20232 . 15382234

[32] Motlagh HN, Wrabl JO, Li J, Hilser VJ. The ensemble nature of allostery. Nature. 2014;508(7496):331–339. 10.1038/nature13001 . 24740064 PMC4224315

[33] Reichheld SE, Yu Z, Davidson AR. The induction of folding cooperativity by ligand binding drives the allosteric response of tetracycline repressor. Proc Natl Acad Sci U S A. 2009;106(52):22263–22268. 10.1073/pnas.0911566106 . 20080791 PMC2799725

[34] Resch M, Striegl H, Henssler EM, Sevvana M, Egerer-Sieber C, Schiltz E, et al A protein functional leap: how a single mutation reverses the function of the transcription regulator TetR. Nucleic Acids Res. 2008;36(13):4390–4401. 10.1093/nar/gkn400 . 18587152 PMC2490752

[35] Sevvana M, Goetz C, Goeke D, Wimmer C, Berens C, Hillen W, et al An exclusive alpha/beta code directs allostery in TetR-peptide complexes. J Mol Biol. 2012;416(1):46–56. 10.1016/j.jmb.2011.12.008 . 22178479

[36] Laemmli UK. Cleavage of structural proteins during the assembly of the head of bacteriophage T4. Nature. 1970;227(5259):680–685. . 5432063 10.1038/227680a0

[37] Mueller U, Darowski N, Fuchs MR, Forster R, Hellmig M, Paithankar KS, et al Facilities for macromolecular crystallography at the Helmholtz-Zentrum Berlin. J Synchrotron Radiat. 2012;19(Pt 3):442–449. 10.1107/S0909049512006395 . 22514183 PMC3408958

[38] Kabsch W. XDS. Acta Crystallogr D Biol Crystallogr. 2010;66(Pt 2):125–132. 10.1107/S0907444909047337 . 20124692 PMC2815665

[39] Karplus PA, Diederichs K. Linking crystallographic model and data quality. Science. 2012;336(6084):1030–1033. 10.1126/science.1218231 . 22628654 PMC3457925

[40] McCoy AJ. Solving structures of protein complexes by molecular replacement with Phaser. Acta Crystallogr D Biol Crystallogr. 2007;63(Pt 1):32–41. 10.1107/S0907444906045975 . 17164524 PMC2483468

[41] Emsley P, Cowtan K. Coot: model-building tools for molecular graphics. Acta Crystallogr D Biol Crystallogr. 2004;60(Pt 12 Pt 1):2126–2132. 10.1107/S0907444904019158 . 15572765

[42] Adams PD, Afonine PV, Bunkoczi G, Chen VB, Davis IW, Echols N, et al PHENIX: a comprehensive Python-based system for macromolecular structure solution. Acta Crystallogr D Biol Crystallogr. 2010;66(Pt 2):213–221. 10.1107/S0907444909052925 . 20124702 PMC2815670

[43] Lebedev AA, Young P, Isupov MN, Moroz OV, Vagin AA, Murshudov GN. JLigand: a graphical tool for the CCP4 template-restraint library. Acta Crystallogr D Biol Crystallogr. 2012;68(Pt 4):431–440. 10.1107/S090744491200251X . 22505263 PMC3322602

[44] Chen VB, Arendall WB 3rd, Headd JJ, Keedy DA, Immormino RM, Kapral GJ, et al MolProbity: all-atom structure validation for macromolecular crystallography. Acta Crystallogr D Biol Crystallogr. 2010;66(Pt 1):12–21. 10.1107/S0907444909042073 . 20057044 PMC2803126

[45] Pettersen EF, Goddard TD, Huang CC, Couch GS, Greenblatt DM, Meng EC, et al UCSF Chimera—a visualization system for exploratory research and analysis. J Comput Chem. 2004;25(13):1605–1612. 10.1002/jcc.20084 . 15264254

[46] Laskowski RA, Swindells MB. LigPlot+: multiple ligand-protein interaction diagrams for drug discovery. J Chem Inf Model. 2011;51(10):2778–2786. 10.1021/ci200227u . 21919503

[47] Kabsch W. A solution for the best rotation to relate two sets of vectors. Acta Crystallographica Section A. 1976;32(5):922–923. 10.1107/s0567739476001873

[48] Winn MD, Ballard CC, Cowtan KD, Dodson EJ, Emsley P, Evans PR, et al Overview of the CCP4 suite and current developments. Acta Crystallogr D Biol Crystallogr. 2011;67(Pt 4):235–242. 10.1107/S0907444910045749 . 21460441 PMC3069738

[49] Dundas J, Ouyang Z, Tseng J, Binkowski A, Turpaz Y, Liang J. CASTp: computed atlas of surface topography of proteins with structural and topographical mapping of functionally annotated residues. Nucleic Acids Res. 2006;34(Web Server issue):W116–118. 10.1093/nar/gkl282 . 16844972 PMC1538779

[50] Sali A, Blundell TL. Comparative Protein Modeling by Satisfaction of Spatial Restraints. Journal of Molecular Biology. 1993;234(3):779–815. 10.1006/jmbi.1993.1626 . 8254673

[51] Jorgensen WL, Chandrasekhar J, Madura JD, Impey RW, Klein ML. Comparison of Simple Potential Functions for Simulating Liquid Water. Journal of Chemical Physics. 1983;79(2):926–935. 10.1063/1.445869 .

[52] Pronk S, Pall S, Schulz R, Larsson P, Bjelkmar P, Apostolov R, et al GROMACS 4.5: a high-throughput and highly parallel open source molecular simulation toolkit. Bioinformatics. 2013;29(7):845–854. 10.1093/bioinformatics/btt055 . 23407358 PMC3605599

[53] Hornak V, Abel R, Okur A, Strockbine B, Roitberg A, Simmerling C. Comparison of multiple amber force fields and development of improved protein backbone parameters. Proteins-Structure Function and Bioinformatics. 2006;65(3):712–725. 10.1002/prot.21123 . PMC4805110 16981200

[54] Lindorff-Larsen K, Piana S, Palmo K, Maragakis P, Klepeis JL, Dror RO, et al Improved side-chain torsion potentials for the Amber ff99SB protein force field. Proteins-Structure Function and Bioinformatics. 2010;78(8):1950–1958. 10.1002/prot.22711 . PMC2970904 20408171

[55] Perez A, Marchan I, Svozil D, Sponer J, Cheatham TE, Laughton CA, et al Refinenement of the AMBER force field for nucleic acids: Improving the description of alpha/gamma conformers. Biophysical Journal. 2007;92(11):3817–3829. 10.1529/biophysj.106.097782 . 17351000 PMC1868997

[56] Bussi G, Donadio D, Parrinello M. Canonical sampling through velocity rescaling. Journal of Chemical Physics. 2007;126(1). doi: Artn 014101 10.1063/1.2408420 . 17212484

[57] Parrinello M, Rahman A. Polymorphic Transitions in Single-Crystals—a New Molecular-Dynamics Method. Journal of Applied Physics. 1981;52(12):7182–7190. 10.1063/1.328693 .

[58] Darden T, York D, Pedersen L. Particle Mesh Ewald—an N.Log(N) Method for Ewald Sums in Large Systems. Journal of Chemical Physics. 1993;98(12):10089–10092. 10.1063/1.464397 .

[59] Sievers F, Wilm A, Dineen D, Gibson TJ, Karplus K, Li W, et al Fast, scalable generation of high-quality protein multiple sequence alignments using Clustal Omega. Mol Syst Biol. 2011;7:539 10.1038/msb.2011.75 . 21988835 PMC3261699

[60] Holm L, Rosenstrom P. Dali server: conservation mapping in 3D. Nucleic Acids Res. 2010;38(Web Server issue):W545–549. 10.1093/nar/gkq366 . 20457744 PMC2896194

