# Supplementary material for: Crystal Structures of the Global Regulator DasR from Streptomyces coelicolor: Implications for the Allosteric Regulation of GntR/HutC Repressors
Source: PLoS One. 2016 Jun 23;11(6):e0157691. doi: 10.1371/journal.pone.0157691 (PMC4918961; doi:10.1371/journal.pone.0157691)
Supplement: S1 Fig — The stereo views show the interaction of DasR-EBD with the β-anomeric configuration of (a) GlcN-6-P and (b) GlcNAc-6-P. GlcN-6-P, GlcNAc-6-P and the interacting protein residues are presented as stick models and water molecules are depicted as red spheres. (PDF) [file pone.0157691.s001.pdf]

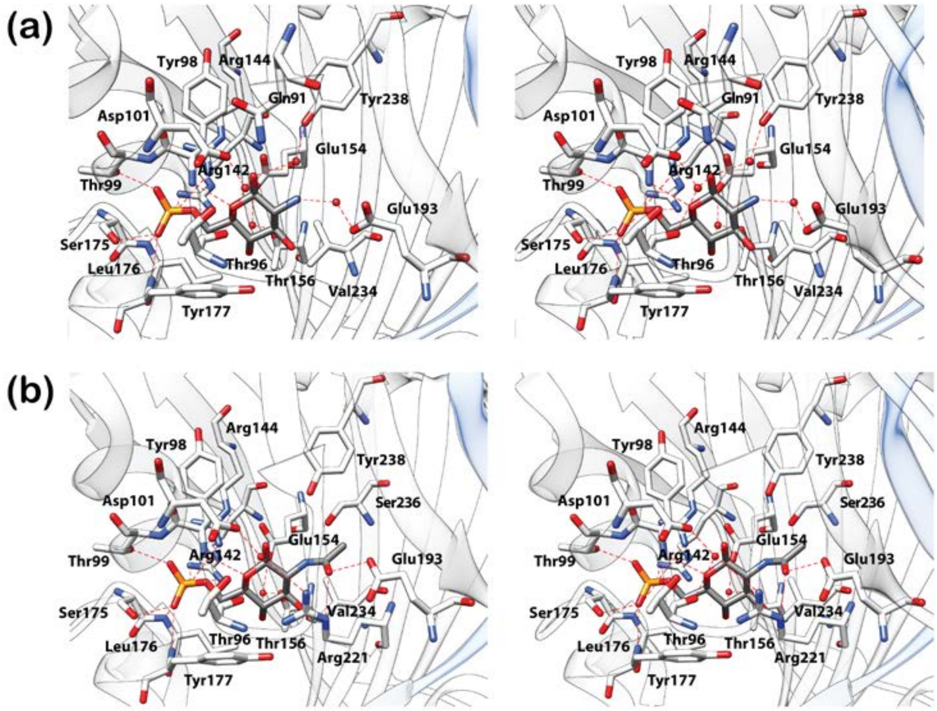

S1 Fig. Closeup view of the effector-binding site of DasR-EBD. The stereo views show the interaction of DasR-EBD with the β-anomeric configuration of (a) GlcN-6-P and (b) GlcNAc-6-P. GlcN-6-P, GlcNAc-6-P and the interacting protein residues are presented as stick models and water molecules are depicted as red spheres. In the sugar molecules, the phosphor, oxygen, nitrogen and carbon atoms are coloured in yellow, red, dark blue and grey, respectively.
