# Supplementary material for: Crystal Structures of the Global Regulator DasR from Streptomyces coelicolor: Implications for the Allosteric Regulation of GntR/HutC Repressors
Source: PLoS One. 2016 Jun 23;11(6):e0157691. doi: 10.1371/journal.pone.0157691 (PMC4918961; doi:10.1371/journal.pone.0157691)
Supplement: S2 Fig — Simulated annealing Fo-Fc OMIT maps show the GlcN-6-P-binding site of (a) chain A and (b) chain B as well as the GlcNAc-6-P-binding site of (c) chain A and (d) chain B in the respective effector-bound structures of DasR-EBD. Maps were calculated with PHENIX and are contoured as green mesh at 3.0 σ. Sugar molecules and all protein residues omitted during refinement and map calculation are shown as stick models. Water molecules are presented as red spheres. (e) and (f) Magnification of GlcNAc-6-P in (e) chain A and (f) chain B showing the corresponding simulated annealing Fo-Fc OMIT maps to illustrate the unambiguity of the ligand position and its anomeric configuration. (PDF) [file pone.0157691.s002.pdf]

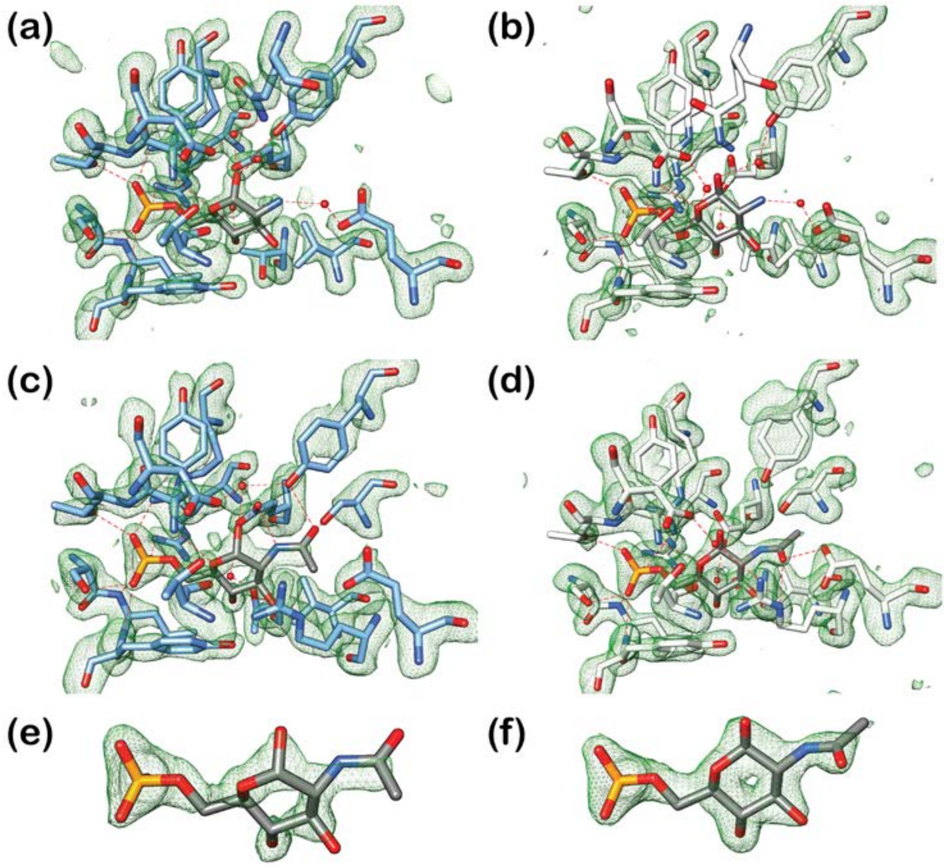

S2 Fig. Electron density maps of the effector-binding site of DasR-EBD with GlcN-6-P and GlcNAc-6-P. Simulated annealing F_o-F_c OMIT maps show the GlcN-6-P-binding site of (a) chain A and (b) chain B as well as the GlcNAc-6-P-binding site of (c) chain A and (d) chain B in the respective effector-bound structures of DasR-EBD. Maps were calculated with PHENIX and are contoured as green mesh at 3.0 σ. Sugar molecules and all protein residues omitted during refinement and map calculation are shown as stick models. Water molecules are presented as red spheres. (e) and (f) Magnification of GlcNAc-6-P in (e) chain A and (f) chain B showing the corresponding simulated annealing F_o-F_c OMIT maps to illustrate the unambiguity of the ligand position and its anomeric configuration. In the sugar molecules, the phosphor, oxygen, nitrogen and carbon atoms are coloured in yellow, red, dark blue and grey, respectively.
