# Supplementary material for: Crystal Structures of the Global Regulator DasR from Streptomyces coelicolor: Implications for the Allosteric Regulation of GntR/HutC Repressors
Source: PLoS One. 2016 Jun 23;11(6):e0157691. doi: 10.1371/journal.pone.0157691 (PMC4918961; doi:10.1371/journal.pone.0157691)
Supplement: S3 Fig — A snapshot from a simulation (a) with a corresponding zoomed view (c), as well as the crystal structure (b) with a corresponding zoomed view (d) is shown. Selected regions in (a) and (c) were coloured as in Fig 8. Specific residues are illustrated as stick model. Residue Glu196 originates from the neighbouring chain of the biological dimer. (PDF) [file pone.0157691.s003.pdf]

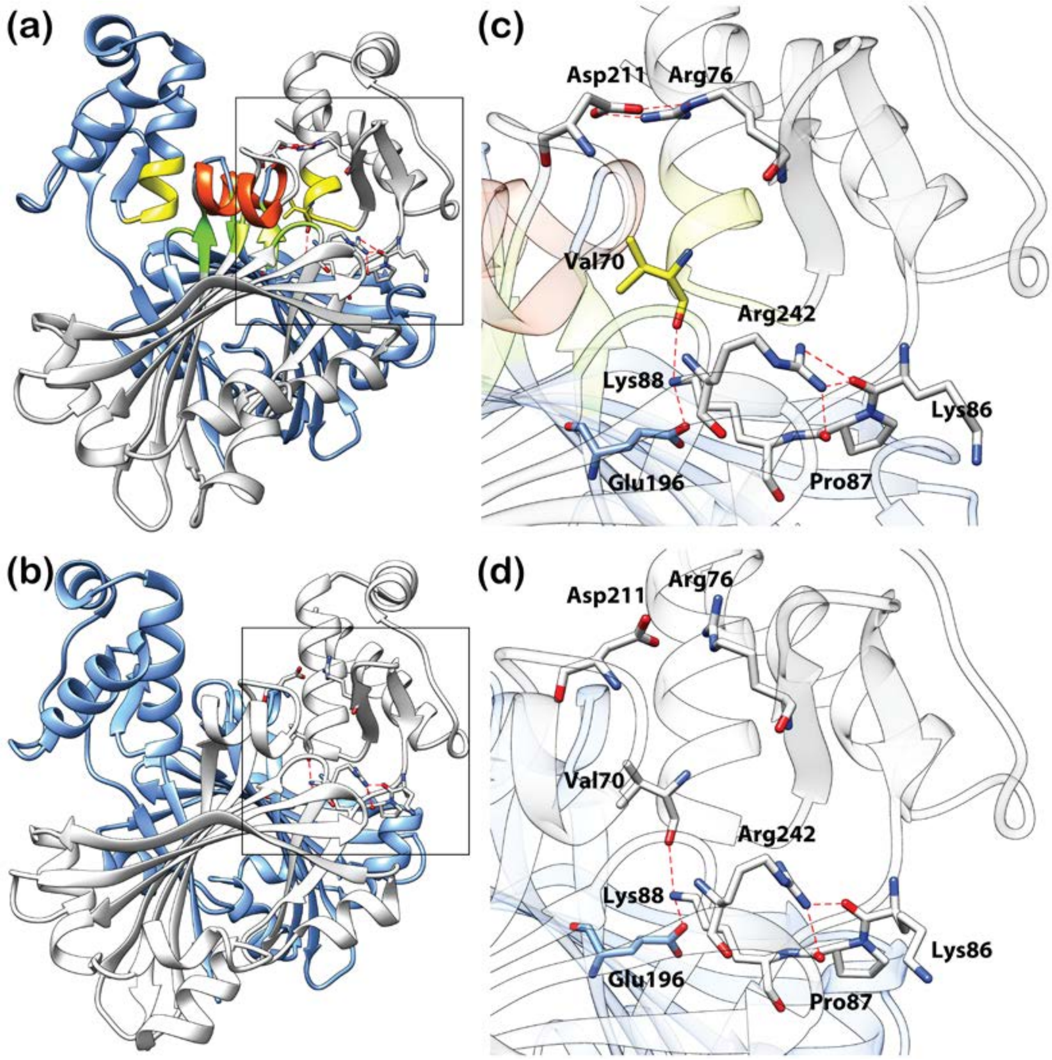

S3 Fig. Distinct hydrogen bonds keep the DBDs of DasR in an 'upwards' position. A snapshot from a simulation (a) with a corresponding zoomed view (c), as well as the crystal structure (b) with a corresponding zoomed view (d) is shown. Selected regions in (a) and (c) were coloured as in Fig. 8. Specific residues are illustrated as stick model. Residue Glu196 originates from the neighbouring chain of the biological dimer.
