# Supplementary material for: Crystal Structures of the Global Regulator DasR from Streptomyces coelicolor: Implications for the Allosteric Regulation of GntR/HutC Repressors
Source: PLoS One. 2016 Jun 23;11(6):e0157691. doi: 10.1371/journal.pone.0157691 (PMC4918961; doi:10.1371/journal.pone.0157691)
Supplement: S4 Fig — The sequence alignment was performed with CLUSTAL OMEGA [59] using the canonical protein sequences of the structurally characterized (full or partial) GntR/HutC family members specified in Supplemental S2 Table. Secondary structure elements refer to the topology of DasR and are marked with (h) or (s) for α-helices and β-strands, respectively. For a detailed classification, the familiar nomenclature αD/E and βD/E is used. Residues involved in DNA binding in NagR [11] and effector binding in DasR or NagR are highlighted by a coloured background. Residues involved in of DNA and effector binding, e.g. those forming base-specific contacts with the DNA, or hydrogen bonds as well as hydrophobic and CH/π interactions with the phosphorylated sugar, are additionally marked by a black arrow. (PDF) [file pone.0157691.s004.pdf]

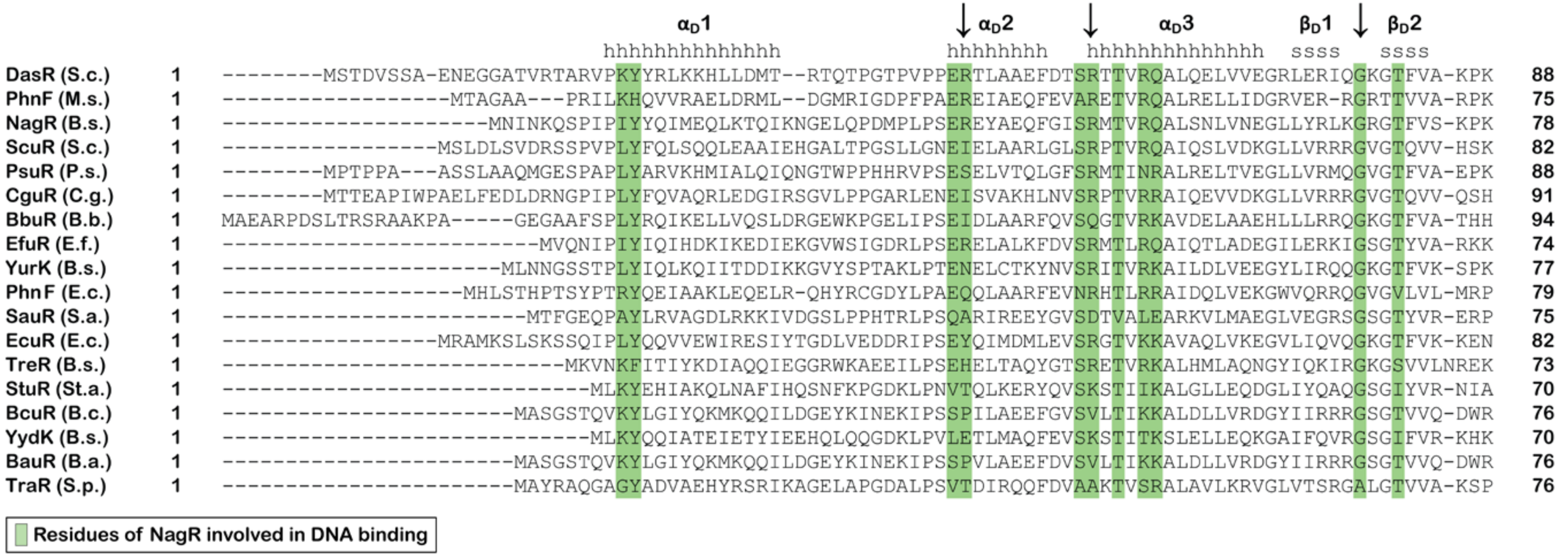

S4 Fig. Sequence alignment of structurally characterized members of the GntR/HutC transcription factor family. The sequence alignment was performed with CLUSTAL OMEGA [59] using the canonical protein sequences of the structurally characterized (full or partial) GntR/HutC family members specified in Supplemental Table S2. Secondary structure elements refer to the topology of DasR and are marked with (h) or (s) for α-helices and β-strands, respectively. For a detailed classification, the familiar nomenclature $\alpha_{D/E}$ and $\beta_{D/E}$ is used. Residues involved in DNA binding in NagR [11] and effector binding in DasR or NagR are highlighted by a coloured background. Residues involved in of DNA and effector binding, e.g. those forming base-specific contacts with the DNA, or hydrogen bonds as well as hydrophobic and CH/π interactions with the phosphorylated sugar, are additionally marked by a black arrow.

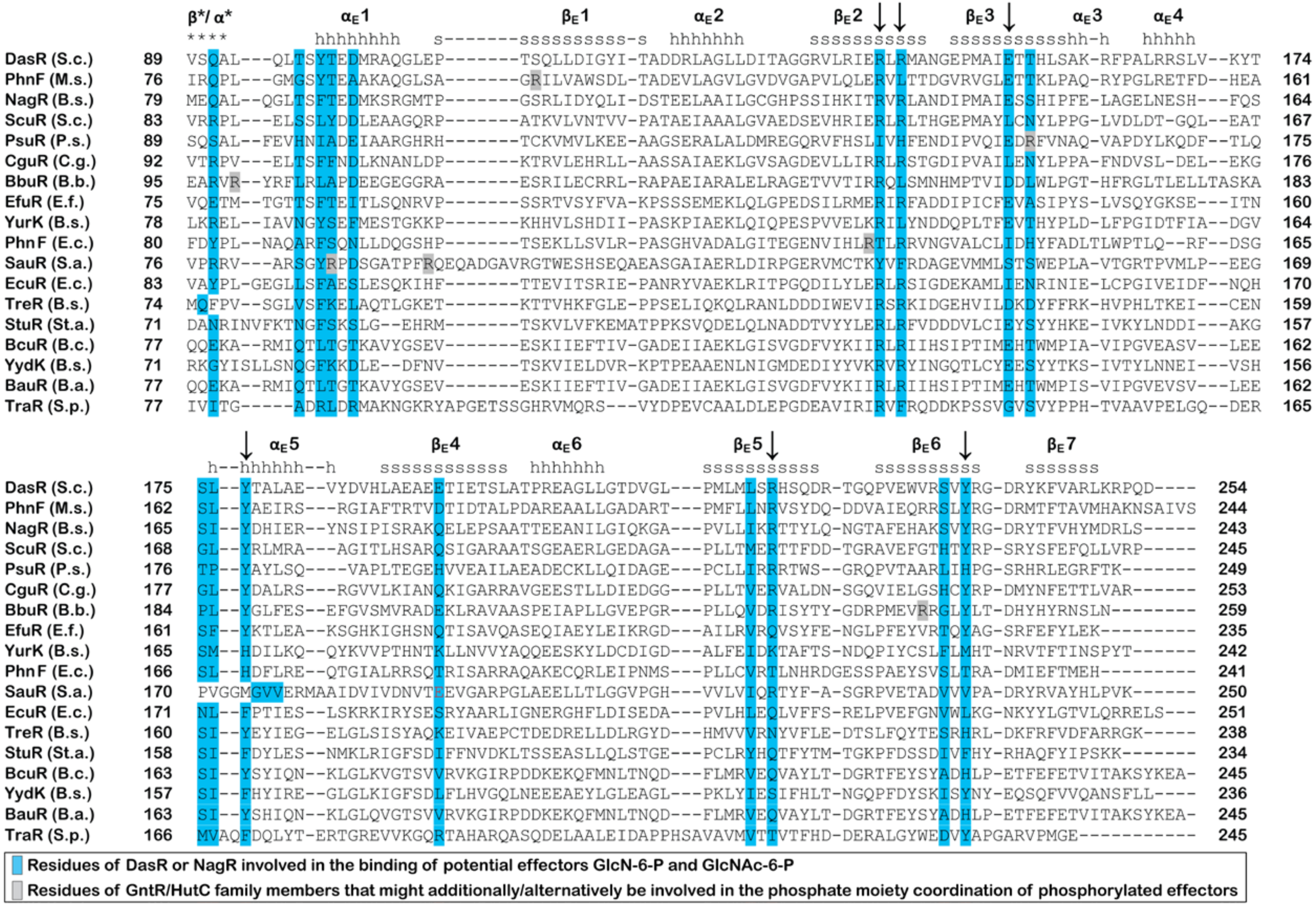

S4 Fig. Sequence alignment of structurally characterized members of the GntR/HutC transcription factor family (continued)
