# Supplementary material for: Crystal Structures of the Global Regulator DasR from Streptomyces coelicolor: Implications for the Allosteric Regulation of GntR/HutC Repressors
Source: PLoS One. 2016 Jun 23;11(6):e0157691. doi: 10.1371/journal.pone.0157691 (PMC4918961; doi:10.1371/journal.pone.0157691)
Supplement: S5 Fig — The sequence alignment was performed with CLUSTAL OMEGA [59] using the canonical protein sequences of entries Q9K492 and O34817 from the UniProt database [19]. Secondary structure elements refer to the topology of DasR and are marked with (h) or (s) for α-helices and β-strands, respectively. For a detailed classification, the familiar nomenclature αE and βE is used. Residues of DasR or NagR involved in effector binding (as identified with LIGPLOT+ [46]) are highlighted by a blue-coloured background. If these amino acids are fully conserved in both transcription factors, they are additionally displayed in bold red letters. In general, fully conserved residues in both sequences are marked with an asterisk (*), while the conservation between groups of strongly and weakly similar properties is labelled with a colon (:) and a period (.), respectively. (PDF) [file pone.0157691.s005.pdf]

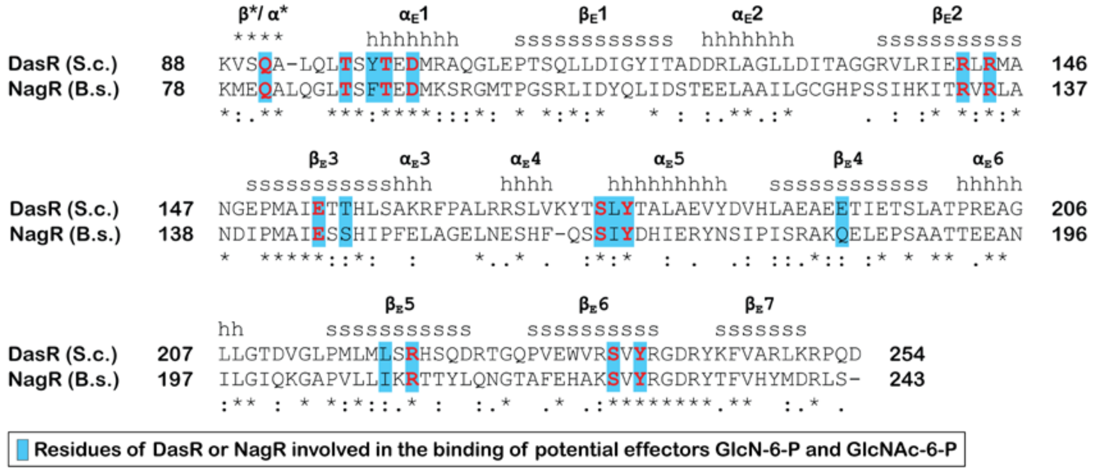

S5 Fig. Sequence alignment of DasR-EBD from *S. coelicolor* and NagR-EBD from *B. subtilis*. The sequence alignment was performed with CLUSTAL OMEGA [59] using the canonical protein sequences of entries Q9K492 and O34817 from the UniProt database [19]. Secondary structure elements refer to the topology of DasR and are marked with (h) or (s) for α-helices and β-strands, respectively. For a detailed classification, the familiar nomenclature α_E and β_E is used. Residues of DasR or NagR involved in effector binding (as identified with LIGPLOT+ [46]) are highlighted by a blue-coloured background. If these amino acids are fully conserved in both transcription factors, they are additionally displayed in bold red letters. In general, fully conserved residues in both sequences are marked with an asterisk (*), while the conservation between groups of strongly and weakly similar properties is labelled with a colon (:) and a period (.), respectively.
