# Supplementary material for: Crystal Structures of the Global Regulator DasR from Streptomyces coelicolor: Implications for the Allosteric Regulation of GntR/HutC Repressors
Source: PLoS One. 2016 Jun 23;11(6):e0157691. doi: 10.1371/journal.pone.0157691 (PMC4918961; doi:10.1371/journal.pone.0157691)
Supplement: S6 Fig — (a) and (b) Superposition of the crystal structures of ligand-free SauR from S. avermitilis (grey, PDB-ID 3EET), ligand-free DasR from S. coelicolor (blue, PDB-ID 4ZS8), GlcNAc-6-P-bound NagR from B. subtilis (green, PDB-ID 4U0W) and ligand-free PhnF from M. smegmatis ((khaki, PDB-ID 3F8M) displayed as a cartoon representation in a side view (a) and a top view (b). The ligand GlcNAc-6-P bound to NagR is shown as a stick model. The EBDs of all four dimeric structures were rendered transparent for clarity. (PDF) [file pone.0157691.s006.pdf]

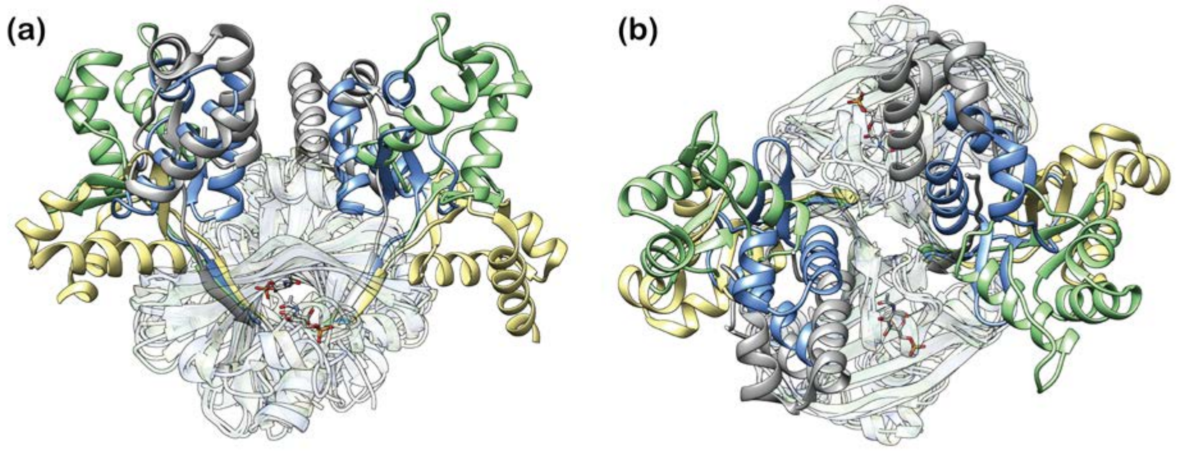

S6 Fig. Remaining DBD flexibility among GntR/HutC transcription factors with β-strand β* formed in the linker segment. (a) and (b) Superposition of the crystal structures of ligand-free SauR from *S. avermitilis* (grey, PDB-ID 3EET), ligand-free DasR from *S. coelicolor* (blue, PDB-ID 4ZS8), GlcNAc-6-P-bound NagR from *B. subtilis* (green, PDB-ID 4U0W) and ligand-free PhnF from *M. smegmatis* (light yellow, PDB-ID 3F8M) displayed as a cartoon representation in a side view (a) and a top view (b). The ligand GlcNAc-6-P bound to NagR is shown as a stick model. The EBDs of all four dimeric structures were rendered transparent for clarity.
