# Supplementary material for: Crystal Structures of the Global Regulator DasR from Streptomyces coelicolor: Implications for the Allosteric Regulation of GntR/HutC Repressors
Source: PLoS One. 2016 Jun 23;11(6):e0157691. doi: 10.1371/journal.pone.0157691 (PMC4918961; doi:10.1371/journal.pone.0157691)
Supplement: S1 Table — The pocket volumes were calculated as described in the ‘Materials and Methods’ section. (PDF) [file pone.0157691.s007.pdf]

S1 Table. Comparison of the effector-binding sites (pockets) from the crystal structures of ligand-free DasR-EBD, ligand-free DasR and effector-bound DasR-EBD. The pocket volumes were calculated as described in the 'Materials and Methods' section.

**Structure**	**ligand-free DasR-EBD**	**ligand-free DasR**		**DasR-EBD + GlcN-6-P**		**DasR-EBD + GlcNAc-6-P**	
chain-ID (effector configuration)	A (-)	A (-)	B (-)	A (α)	B (β)	A (α)	B (β)
MS volume [a] (Å^3)	1029.5	1126.8	1084.8	576.6	668.3	577.9	693.1
SA volume [b] (Å^3)	**218.0**	**252.0**	**271.3**	**74.7**	**94.1**	**94.9**	**101.8**
pocket MS area [c] (Å^2)	756.5	874.8	804.0	577.6	633.7	488.7	643.0
pocket SA area [d] (Å^2)	426.8	456.1	422.1	196.5	238.0	217.8	251.1
# openings [e]	**2**	**2**	**2**	**0**	**1**	**0**	**1**
mouth MS area [f] (Å^2)	146.9	157.3	160.6	-	12.1	-	28.1
mouth SA area [g] (Å^2)	33.5	51.0	58.4	-	0.3	-	4.6
MS circumference [h] (Å)	89.8	86.0	83.9	-	12.7	-	22.1
SA circumference [i] (Å)	73.1	67.0	65.5	-	4.7	-	13.7

pocket volume based on the [a] molecular surface / [b] solvent-accessible surface
[c] pocket molecular surface area / [d] pocket solvent-accessible surface area
[e] number of mouths or openings to the external molecular surface
total area of mouth opening(s) based on the [f] molecular surface / [g] solvent-accessible surface
total circumference of mouth opening(s) based on the [h] molecular surface / [i] solvent-accessible surface
