# Supplementary material for: Crystal Structures of the Global Regulator DasR from Streptomyces coelicolor: Implications for the Allosteric Regulation of GntR/HutC Repressors
Source: PLoS One. 2016 Jun 23;11(6):e0157691. doi: 10.1371/journal.pone.0157691 (PMC4918961; doi:10.1371/journal.pone.0157691)
Supplement: S2 Table — The listed regulators were identified by a protein structure database search (Dali Lite v.3, [60]) via the Dali server using the crystal structure of full-length DasR (PDB-ID 4ZS8) as a search model. From the resulting structures only those containing a GntR-family-specific wHTH domain as well as a HutC-subfamily-specific UTRA domain (as described in the respective entry in the UniProt database [19]) were used for a subsequent multiple sequence alignment via CLUSTAL OMEGA [59] that is shown in S4 Fig. For a better discrimination, regulators without an individual gene or protein name and mostly of unknown function were given unambiguous acronyms, e.g. ScuR for S. coelicolor unnamed Regulator. The panel “Match of characteristic residues” describes residues that are explicitly involved in DNA binding of NagR [11] and in effector binding of DasR and/or NagR (Fig 9), and their equivalents from other GntR/HutC family members inferred by structural comparison with DasR and NagR. Residues marked in red were only inferred by sequence due to the lack of structural information. (PDF) [file pone.0157691.s008.pdf]

**S2 Table. Structurally characterized members of the GntR/HutC transcription factor family.** The listed regulators were identified by a protein structure database search (Dali Lite v.3, [60]) *via* the Dali server using the crystal structure of full-length DasR (PDB-ID 4ZS8) as a search model. From the resulting structures only those containing a GntR-family-specific wHTH domain as well as a HutC-subfamily-specific UTRA domain (as described in the respective entry in the UniProt database [19]) were used for a subsequent multiple sequence alignment *via* CLUSTAL OMEGA [59] that is shown in S4 Fig. For a better discrimination, regulators without an individual gene or protein name and mostly of unknown function were given unambiguous acronyms, e.g. ScuR for *S. coelicolor* unnamed Regulator. The panel "Match of characteristic residues" describes residues that are explicitly involved in DNA binding of NagR [11] and in effector binding of DasR and/or NagR (Fig 9), and their equivalents from other GntR/HutC family members inferred by structural comparison with DasR and NagR. Residues marked in red were only inferred by sequence due to the lack of structural information.

| Name | Organism | Gene | Uniprot ID<br>PDB-ID | Function | Residues in structure (total) | Sequence identity to DasR (%) | Match of characteristic residues | | Additional information |
|---|---|---|---|---|---|---|---|---|---|
| | | | | | | | DBD | EBD | |
| DasR | *Streptomyces coelicolor* | *dasR* | Q9K492<br>4ZS8 | global regulator; part of nutrient sensing system | 22-251<br>(254) | 100 | R48 R58 G79 | R142 R144 E154 Y177 R221 Y238 | |
| PhnF | *Mycobacterium smegmatis* | *phnF* | A0QQ72<br>3F8M | transcr. regulator of unknown function | 2-244<br>(244) | 39.6 | R36 R46 G66 | R129 L131 E141 Y164 R207 Y224 | S84 & R89 for phosphate moiety interaction [18] |
| NagR | *Bacillus subtilis* | *yvoA* | O34817<br>4U0V | local regulator; part of nutrient sensing system | 1-243<br>(243) | 38.8 | R38 R48 G69 | R133 R135 E145 Y167 R211 Y228 | |
| ScuR | *Streptomyces coelicolor* | *SCO6256* | Q9RKT6<br>2RA5 | transcr. regulator of unknown function | 1-245<br>(245) | 34.6 | I42 R52 G73 | R136 R138 L148 Y170 R213 Y230 | S,R meso-tartaric acid in binding pocket |
| PsuR | *Pseudomonas syringae* | *hutC* | Q87UX0<br>2PKH | repressor of histidine utilization | 105-249<br>(249) | 30.5 | S48 R58 G79 | I143 H145 E155 Y178 R220 H236 | R156 for phosphate moiety interaction? |

| | | | | | | | | |
|---|---|---|---|---|---|---|---|---|
| CguR | *Corynebacterium glutamicum* | *cg0196* | Q8NTZ4 2P19 | transcr. regulator of unknown function | 105-253 (253) | 30.4 | I51 R61 G82 | R145 R147 L157 Y177 R222 Y239 | |
| BbuR | *Bacillus bronchiseptica* | *BB3683* | Q7WD95 3CNV | transcr. regulator of unknown function | 98-259 (259) | 29.8 | I54 Q64 G85 | R149 L151 D161 Y186 R230 Y247 | R99 & R243 for phosphate moiety interaction? Citrate anion in binding pocket |
| EfuR | *Enterococcus faecalis* | *EF1328* | Q835P8 3DDV | transcr. regulator of unknown function | 91-235 (235) | 28.9 | R34 R44 G65 | R128 R130 E141 F163 Q207 L224 | |
| YurK | *Bacillus subtilis* | *yurK* | O32152 2IKK | transcr. regulator of unknown function | 94-242 (242) | 26.4 | N37 R47 G68 | R132 L134 E144 H167 K211 M228 | Sulphate anion in binding pocket |
| PhnF | *Escherichia coli* | *phnF* | K16684 2FA1 | transcr. regulator of unknown function | 85-241 (241) | 25.3 | Q39 R49 G70 | T134 R136 I146 H168 R212 T230 | R133 for phosphate moiety interaction? |
| SauR | *Streptomyces avermitilis* | *SAV3189* | Q82IF8 3EET | transcr. regulator of unknown function | 1-250 (250) | 22.9 | A35 D45 G66 | Y137 F139 S149 V177 R220 V236 | R36 for DNA interaction? R86 & R95 for phosphate moiety interaction? |
| EcuR | *Escherichia coli* | *c4276* | Q8FCM7 3HFI | transcr. regulator of unknown function | 82-251 (251) | 22.8 | Y42 R52 G73 | R138 R140 I150 F173 Q217 L234 | No dimerization of EBD |
| TreR | *Bacillus subtilis* | *treR* | P39796 2OGG | repressor of trehalose operon | 90-238 (238) | 21.8 | H32 R42 G63 | R128 R130 D140 Y162 N206 H223 | Sulphate anion in binding pocket |
| StuR | *Staphylococcus aureus* | *SA0254* | Q7A7U1 2OOI | transcr. regulator of unknown function | 73-234 (234) | 21.1 | T30 K40 G61 | R126 R128 E138 F160 Q204 F221 | Two ethylene glycol molecules in area of binding pocket |
| YydK | *Bacillus subtilis* | *yydK* | Q45591 3BWG | transcr. regulator of unknown function | 1-236 (236) | 20.4 | E30 K40 G61 | R125 R127 E137 F159 S203 Y220 | |
| BauR | *Bacillus anthracis* | *BA3458* | Q81MW2 3LHE | transcr. regulator of unknown function | 93-232 (245) | 19.3 | P36 V46 G67 | R131 R133 E143 Y165 Q209 H226 | High identity (>97%) to BcuR (*B. cereus*) No dimerization of EBD |
| BcuR | *Bacillus cereus* | *BC3424* | Q735I1 3L5Z | transcr. regulator of unknown function | 92-245 (245) | 18.8 | P36 V46 G67 | R131 R133 E143 Y165 Q209 H226 | High identity (>97%) to BauR (*B. anthracis*) |
| TraR | *Streptomyces phaeochromogenes* | *traR* | Q54677 1V4R | transcr. regulator of unknown function | 7-99 (245) | 16.6 | T36 A46 A66 | R133 R136 G145 Y174 T218 Y235 | NMR structure |
