# Supplementary material for: Crystal Structures of the Global Regulator DasR from Streptomyces coelicolor: Implications for the Allosteric Regulation of GntR/HutC Repressors
Source: PLoS One. 2016 Jun 23;11(6):e0157691. doi: 10.1371/journal.pone.0157691 (PMC4918961; doi:10.1371/journal.pone.0157691)
Supplement: S3 Table — The sequence identities were calculated with CLUSTAL OMEGA [59]. (PDF) [file pone.0157691.s009.pdf]

S3 Table. Pairwise comparison of protein sequence identities between all GntR/HutC transcription factors used in the multiple sequence alignment shown in S4 Fig. The sequence identities were calculated with CLUSTAL OMEGA [59].

Name	Protein sequence identity (%)																	
**DasR**	100																	
**PhnF**	39.6	100																
**NagR**	38.8	31.1	100															
**ScuR**	34.6	24.4	34.6	100														
**PsuR**	30.5	21.7	27.7	26.6	100													
**CguR**	30.4	24.5	28.9	50.0	24.7	100												
**BbuR**	29.8	23.4	26.5	30.4	23.5	31.7	100											
**EfuR**	28.9	24.1	34.0	26.3	22.4	28.9	23.0	100										
**YurK**	26.4	20.5	26.1	24.7	23.5	27.2	24.3	29.4	100									
**PhnF**	25.3	25.4	20.3	27.9	19.1	27.9	23.0	23.3	23.3	100								
**SauR**	22.9	16.0	21.1	22.9	22.7	24.2	22.5	15.9	20.6	17.2	100							
**EcuR**	22.8	19.1	25.9	25.3	21.8	27.5	22.1	26.0	25.2	25.1	19.3	100						
**TreR**	21.8	21.1	24.5	21.8	17.2	19.3	18.8	25.3	19.9	20.0	14.5	21.5	100					
**StuR**	21.1	15.0	21.7	20.6	19.7	19.7	20.9	24.0	22.9	22.1	18.3	19.4	21.2	100				
**YydK**	20.4	16.6	26.6	23.0	16.2	18.8	19.6	25.3	21.6	23.0	15.7	22.6	25.3	39.9	100			
**BauR**	19.3	22.4	22.8	20.6	17.4	22.4	22.3	25.5	20.4	21.8	17.3	18.9	24.9	21.7	24.8	100		
**BcuR**	18.8	22.4	22.8	21.0	18.2	22.8	22.3	25.1	20.4	21.8	17.7	19.3	25.7	22.1	25.2	97.6	100	
**TraR**	16.6	20.5	21.3	21.4	20.2	19.7	19.5	21.5	18.2	18.4	20.9	17.8	15.9	16.7	16.2	19.1	18.7	100
	**DasR**	**PhnF**	**NagR**	**ScuR**	**PsuR**	**CguR**	**BbuR**	**EfuR**	**YurK**	**PhnF**	**SauR**	**EcuR**	**TreR**	**StuR**	**YydK**	**BauR**	**BcuR**	**TraR**
